# Loss of the ER-cargo protein CLN8 increases severity of acute pancreatitis and upregulates ER-stress and ER-phagy

**DOI:** 10.1186/s43556-026-00479-4

**Published:** 2026-06-15

**Authors:** Lukas Zierke, Marcel Gischke, Matthias Sendler, Frank Ulrich Weiss, Silvia Ribback, Dariush Skowronek, Matthias Rath, Henry Völzke, Markus M. Lerch, Ali A. Aghdassi

**Affiliations:** 1 Department of Medicine A, University Medicine Greifswald, Ferdinand-Sauerbruch Str., 17475 Greifswald, Germany; 2 Department of Pathology, University Medicine Greifswald, Greifswald, Germany; 3 Department of Human Genetics, University Medicine and Interfaculty Institute of Genetics and Functional Genomics, University of Greifswald, Greifswald, Germany; 4 Institute for Molecular Medicine, MSH Medical School Hamburg, Hamburg, Germany; 5 Institute for Community Medicine, University Medicine Greifswald, Greifswald, Germany; 6 Ludwig-Maximilians University Munich, Munich, Germany

**Keywords:** Acute pancreatitis, CLN8, Lysosome, Co-localization, Endoplasmic reticulum

## Abstract

Acute pancreatitis is caused by a premature activation of digestive proteases. One hypothesis is based on the proteolytic activation of the serine protease trypsinogen by the lysosomal enzyme cathepsin B (CTSB) after co-localization in the same subcellular compartment. The ER-cargo receptor protein CLN8 (ceroid lipofuscinosis, neuronal) mediates cathepsin transport from the endoplasmic reticulum (ER), the site of enzyme synthesis, to the trans-Golgi system, from which they are distributed to their final destinations. The aim of this study is to investigate the role of CLN8 in acute pancreatitis and intracellular cathepsin trafficking by using isolated pancreatic acinar cells, a CLN8-deficient (*Cln8*^*mnd*^/MsrJ) mouse model, and 266–6 mouse pancreatic acinar tumor cells in which the *Cln8* gene was inactivated by CRISPR/Cas9. Loss of CLN8 mitigated the early phase of acute pancreatitis but did not prevent it completely. We still observed CTSB expression in the endo-lysosomal and secretory compartment albeit enzyme activation was decreased. At later disease stages pancreatic injury increased along with an upregulation of ER-phagy shown by an overexpression of LC3B and the ER-phagy receptor FAM134B as well as autophagolysosome formation and increased ER stress. In summary, our data show that acute pancreatitis still occurs despite disruption of the EGRESS (ER-to-Golgi relaying of enzymes of the lysosomal system) complex implicating alternative intracellular enzyme delivery routes. They also illustrate that ER-stress and ER-phagy aggravate severity at later course of pancreatitis.

**Supplementary Information:**

The online version contains supplementary material available at 10.1186/s43556-026-00479-4.

## Introduction

Acute pancreatitis is a common non-malignant inflammatory gastrointestinal disorder with a complex pathophysiology. One of its hallmarks is a premature and uncontrolled activation of digestive proteases inside pancreatic acinar cells, which starts by the proteolytic cleavage of inactive trypsinogen into its active form [1, 2]. A significant role is ascribed to cathepsin B (CTSB), which is predominantly located in lysosomes, whereas trypsinogen is exclusively found in the secretory granules inside each acinar cell [3]. The co-localization of both enzymes in the same compartment, followed by a CTSB-mediated activation of trypsinogen, is discussed as a potential cause of acute pancreatitis [4, 5]. However, the exact molecular mechanisms of enzyme co-localization are still a matter of debate [6]. 

There are other subcellular compartments with a likely impact on acute pancreatitis. Starting with a prolonged elevation of intracellular Ca^2+^ concentration, both mitochondrial and endoplasmic reticulum (ER-)stress are induced. To circumvent cellular demise caused by ER-stress, autophagy is initiated in pancreatic acinar cells [7–9]. However, an overwhelming stress stimulus can result in cell death and onset of acute pancreatitis. This effect is exemplified by mutations in the cationic trypsinogen gene *PRSS1* leading to misfolding of the protein, induction of ER-stress, and disease onset in humans [10]. In summary, besides protease activation inside the zymogen granules, mitochondrial and endoplasmic reticulum malfunction strongly affects cell damage and severity of pancreatitis.

The ceroid lipofuscinosis protein CLN8 is an ER-associated membrane protein that belongs to the EGRESS (ER-to-Golgi relaying of enzymes of the lysosomal system) complex, having multiple tasks for intact cellular function. It mediates the transport of soluble lysosomal proteins such as cathepsins from the endoplasmic reticulum to the trans-Golgi system [11, 12]. Moreover, mitochondrial metabolism, lysosomal function, Ca^2+^ signaling, and proper cellular morphology are dependent on CLN8 [11, 13–18]. Hence, defects of this protein contribute to the pathogenesis of disorders that are associated with disrupted lysosomal protease activation or mitochondrial function.

In this study, we unveiled the effects of CLN8 on intracellular cathepsin trafficking and initiation of acute pancreatitis. Acute pancreatitis was investigated using a mouse model carrying a mutated *Cln8* gene (*Cln8*^*mnd*^/MsrJ) as well as in vitro in isolated acinar cells and immortal 266–6 murine acinar cells with a CLN8 CRISPR/Cas9 knockout. Despite loss of CLN8, CTSB was still transferred into the secretory compartment, albeit to a lesser extent, and acute pancreatitis was induced. We also noticed increased ER-stress and -phagy in the pancreas when CLN8 was absent. Our findings provide evidence of alternative trafficking mechanism for cathepsins inside the acinar cell and underpin the contributing effect of ER-stress to pancreatic injury in acute pancreatitis.

## Results

### Cathepsin and digestive enzyme expression in CLN8-deficient mice

The absence of CLN8 expression in the B6.KB2-*CLN8*^*mnd*^/MsrJ mice was confirmed by Western Blot analysis of pancreas and liver homogenates (Fig. S1a, b) and immunohistochemical labeling of paraffin-embedded pancreatic tissues (Fig. S1c). Histology appeared normal in CLN8^−/−^ pancreas tissues. The expression of pancreatic proteases was assessed by immunoblot analysis. There were no differences for trypsin, pro- and active forms of cathepsin B (CTSB), cathepsin D (CTSD), and LIMPII expression between CLN8-deficient and wild type mice (Fig. S1a, b). Likewise, measurement of CTSB, pancreatic amylase, lipase, and pre-activated trypsinogen (total trypsin activity in the graph) in pancreas homogenates yielded similar results (Fig. S1d).

### Loss of CLN8 mitigates protease activation in early acute pancreatitis but aggravates injury at late phase

**Fig. 1:**
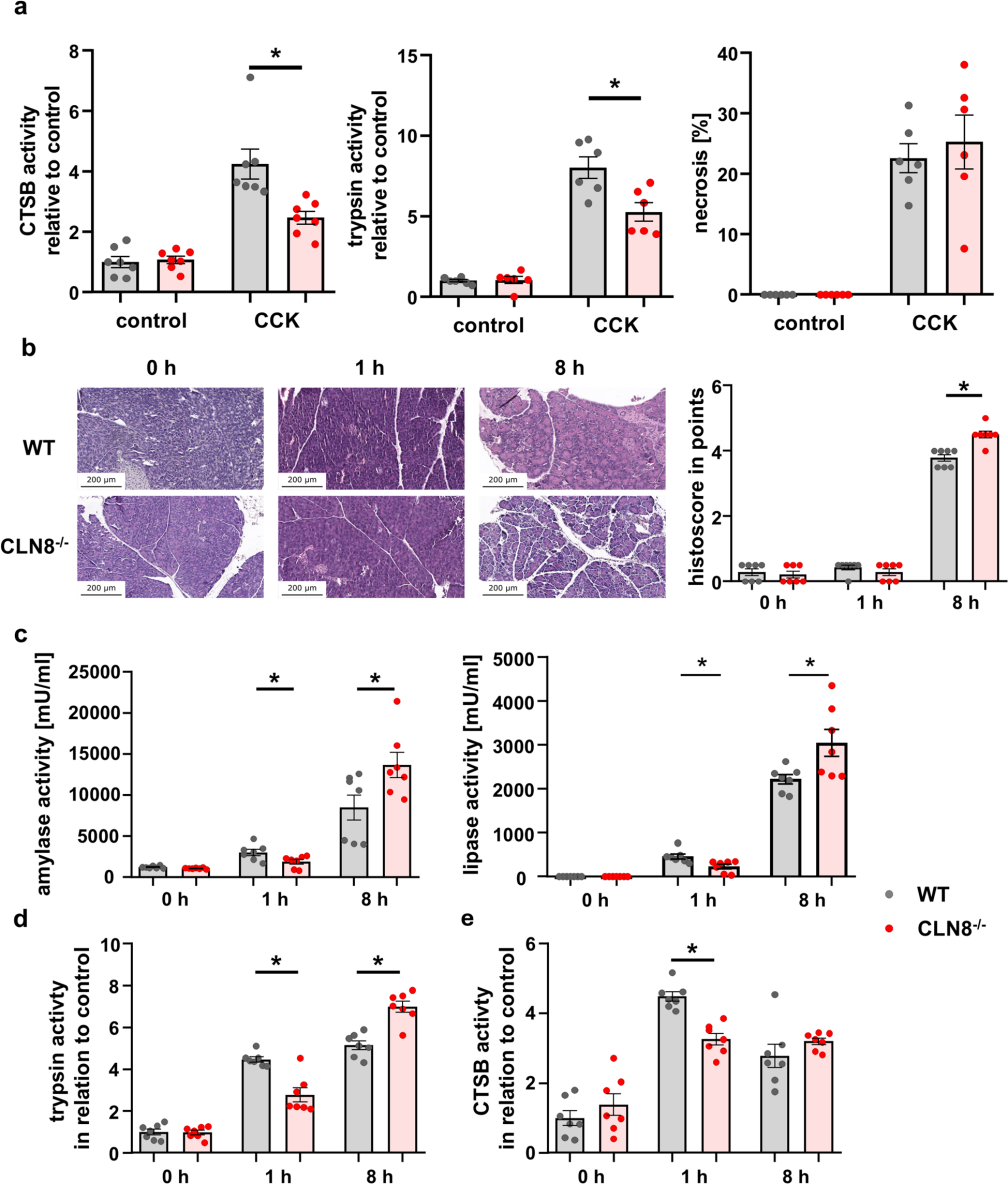
Severity of acute pancreatitis in CLN8^−/−^ mice. **a** CTSB and trypsin activity, measured in isolated living pancreatic acinar cells from wild type mice (WT) and CLN8-deficient mice (CLN8.^−/−^) with and without a supramaximal cholecystokinin stimulation for 30 min, were significantly decreased after CCK-stimulation in the CLN8-deficient cells despite no differences in the necrosis rate. **b** Hematoxylin and eosin stainings of pancreatic tissue of WT and CLN8 knockout mice under physiological conditions (0 h) as well as the early (1 h) and late phase (8 h) of acute pancreatitis showed elevated histological damage in the pancreas of CLN8-deficient mice at the late stage of the disease. Histological scoring of the damage confirmed a significantly increased tissue damage at 8 h in the CLN8 knockout mice. **c**, **d** Amylase and lipase activities in serum (**c**) as well as trypsin (**d**) activity in pancreas homogenates of CLN8-deficient mice transiently decreased during the early phase followed by an increase at 8 h. **e** Similarly, the activity of CTSB in pancreas homogenates was reduced at 1 h but later indicate a trend towards higher activities. At least six animals were used for these experiments and the measurements were performed in triplicates. Values are mean ± SEM. * denotes p < 0.05

Intracellular activities of CTSB and trypsin significantly decreased in CLN8-deficient acinar cells upon supramaximal cholecystokinin (CCK) stimulation, while necrosis remained similar to wild types (Fig. 1a).

To confirm our observations from isolated acinar cells, we continued our experiments using an in vivo model of acute pancreatitis. Local tissue damage, assessed by HE staining and quantification of necrosis, edema, and immune cell infiltration, was similar between both groups at the early disease phase (1 h) but showed more injury at 8 h in CLN8-deficient mice (Fig. 1b). Serum amylase and lipase (Fig. 1c) as well as trypsin activities (Fig. 1d) were lower at 1 h but increased later in the CLN8^−/−^ mice. Activity of CTSB was also lower in pancreas homogenates of CLN8-deficient mice at 1 h but leveled out at 8 h (Fig. 1e). Quantification of apoptotic cells in the pancreas revealed no differences under unstimulated conditions (0 h) but a higher rate at 8 h in CLN8^−/−^ mice (Fig. S2).

In summary, CLN8-deficient mice still developed acute pancreatitis characterized by a reduced protease activity and disease severity immediately after disease onset but significantly increased severity at a late phase.

### Subcellular distribution of cathepsins is not completely disrupted by absence of CLN8

**Fig. 2:**
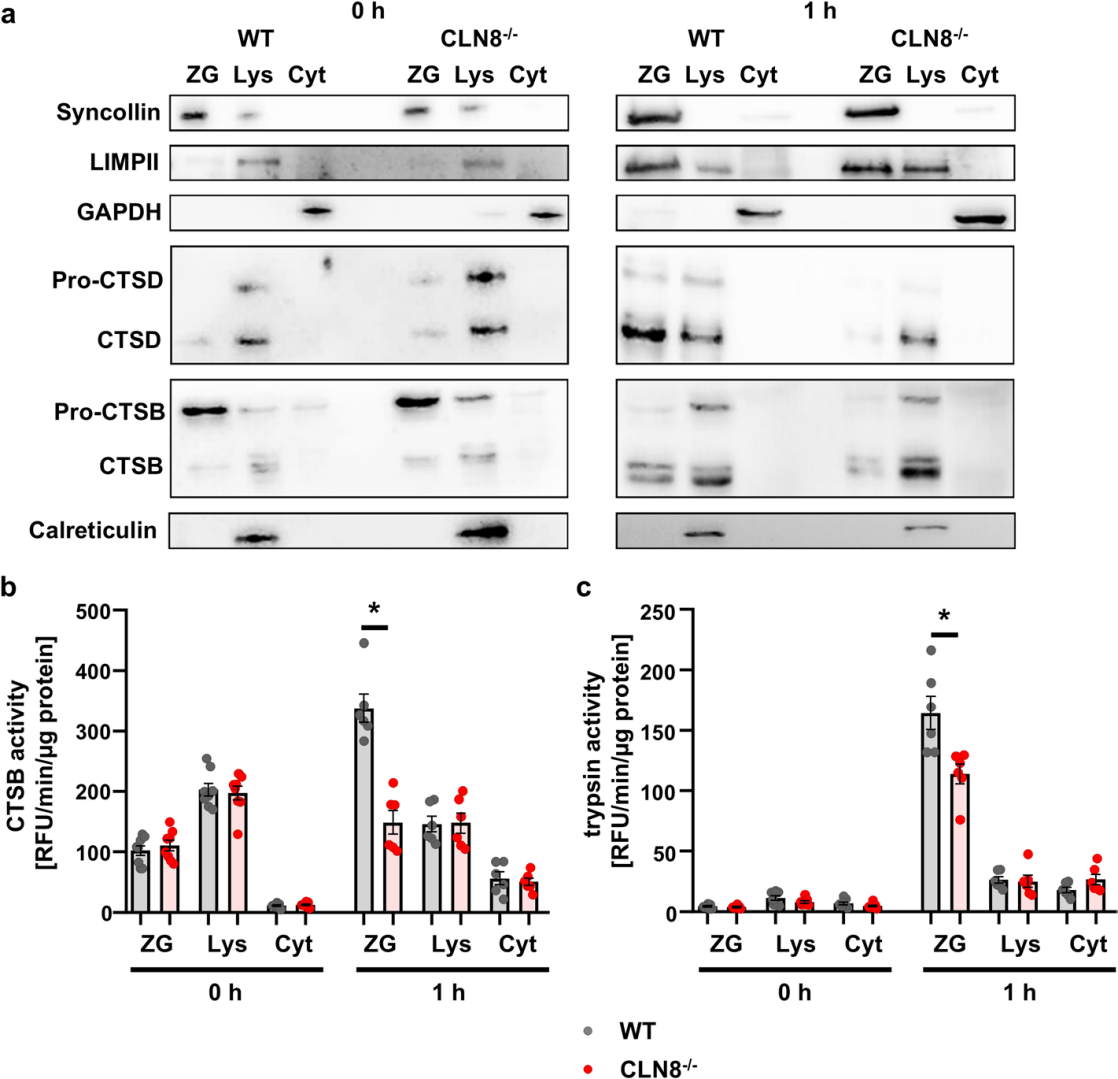
Subcellular cathepsin expression in unstimulated pancreas and after onset of acute pancreatitis CLN8 knockout mice and controls. **a** Immunoblot analysis of subcellular fractions of pancreas homogenates showed a comparable expression of pro- and active forms of cathepsin D (CTSD) and cathepsin B (CTSB) between CLN8-deficient and control mice under unstimulated condition (0 h). While CTSB and CTSD expression shifted towards the zymogen-enriched fraction (ZG) at 1 h in wild types, active CTSB and CTSD were only faintly expressed in the ZG fraction of CLN8 knockout mice. Syncollin, LIMP-2 and GAPDH served as markers for the zymogen-enriched fraction, the lysosome-enriched fraction (Lys), and the cytosolic fraction (Cyt), respectively. The ER-marker calreticulin indicated the presence of ER proteins inside the Lys fraction. **b** Under unstimulated conditions, the subcellular distribution of CTSB activity was independent of CLN8. CCK-stimulation led to a redistribution from the Lys into the ZG fraction, which was distinctly reduced in CLN8-deficient mice. **c** While intrapancreatic trypsin activity was low in normal conditions, caerulein-stimulation led to a remarkable increase of active trypsin in the ZG fraction, which was less pronounced in CLN8-deficient mice. At least six animals were used. The measurements were performed in triplicates. Values are mean ± SEM. * denotes p < 0.05

As CLN8 is an important component for lysosomal enzyme trafficking from the ER to the Golgi [11], we were interested in intracellular cathepsin distribution in the inflamed pancreas after loss of CLN8. Therefore, we prepared subfractionations of pancreas homogenates and measured protein expression and enzyme activities before and after caerulein-stimulation. Differential centrifugation produced three distinct fractions: a heavy, zymogen-granule-enriched fraction (ZG), a lysosome-enriched fraction (Lys), and a cytosolic fraction (Cyt) (Fig. 2a). Syncollin, LIMPII, and GAPDH served as markers for the purity of these fractions. Under physiological conditions, subcellular localizations of the pro- and active forms of CTSD and CTSB were essentially the same between CLN8-deficient and wild type mice. The ER-marker calreticulin was expressed in the lysosomal-enriched fraction, indicating that this fraction not only contains pure lysosomes but also ER-components. After induction of acute pancreatitis in wild types, cathepsin expression redistributed from the lysosomal-enriched into the zymogen-granule-enriched fraction, as reflected by the bands for the active forms of CTSD and CTSB. Likewise, LIMPII expression shifted to the secretory compartment (ZG fraction). In the ZG fraction of CLN8^−/−^ mice bands for active CTSB and CTSD were much weaker. Calreticulin remained exclusively in the lysosomal-enriched fraction indicating that CTSB expression in the zymogen fraction does not result from a contamination of ER-proteins.

**Fig. 3:**
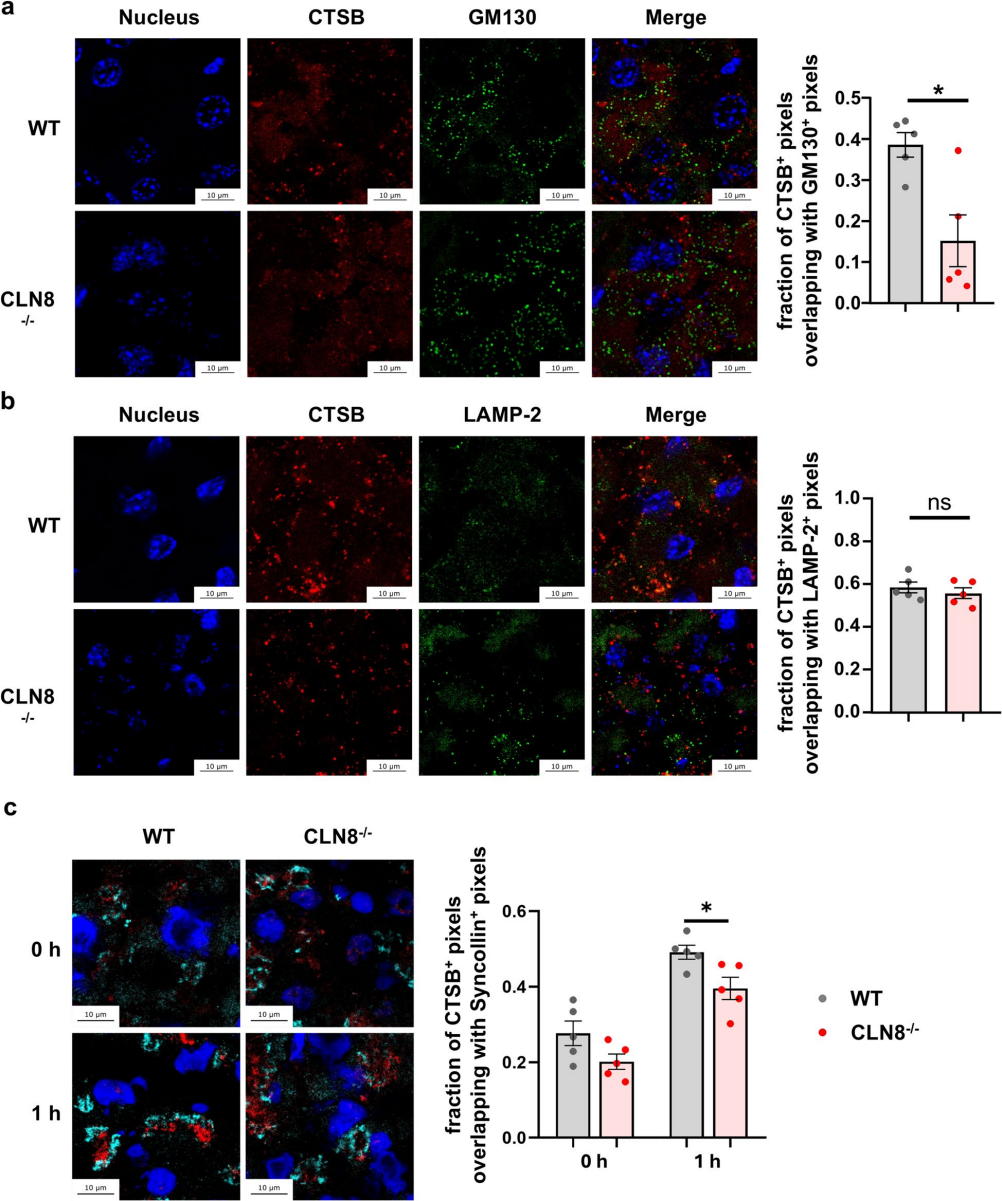
Confocal microscopic imaging of pancreatic tissue of wild type mice (WT) and CLN8-deficient mice (CLN8^−/−^). **a**, **b** Immunofluorescence labeling of paraffin-embedded pancreatic tissue of wild type and CLN8-deficient mice using antibodies against GM130 and LAMP-2 (green), cathepsin B (red), and Sytox Deep Red for labeling of the nucleus (blue) followed by co-localization analysis using Manders split coefficient reflects the intracellular localization of CTSB in the Golgi system. CLN8-deficient mice showed a significantly reduced fraction of CTSB^+^ pixels overlapping with GM130^+^ pixels indicating less amount of CTSB in the Golgi (**a**). The lysosomal localization of CTSB remained intact despite loss of CLN8 as reflected by the fraction of CTSB^+^ pixels overlapping with LAMP-2^+^ pixels according to Manders split coefficient (**b**). **c** Immunofluorescence labeling of cryo-embedded pancreatic tissue of WT and CLN8.^−/−^ mice at 0 h and 1 h acute pancreatitis using antibodies against CTSB (red), syncollin (cyan), and Sytox Deep Red for labeling the nucleus (blue) followed by colocalization analysis using Manders split coefficient showed similar distribution of CTSB at 0 h in both WT and CLN8-deficient pancreas, but a significant decrease of colocalization of CTSB and syncollin after onset of the disease. Visual fields of 5 animals were used for colocalization analysis. Values are mean ± SEM. * denotes p < 0.05

The shift of cathepsin activities into the heavy fraction during the early phase of pancreatitis was quantified by fluorometric measurements (Fig. 2b). Under normal conditions, CTSB activity predominantly accumulated in the lysosomal-enriched compartment and to a lesser extent in the zymogen fraction without any significant differences between CLN8-competent and -deficient mice. As expected, trypsin activity was barely detectable in unstimulated conditions (Fig. 2c). Induction of pancreatitis increased the activities of CTSB and trypsin in the zymogen-granule enriched fractions in both groups although less pronounced in CLN8^−/−^ mice. In contrast, enzyme activities in the lysosomal-enriched and the cytosolic fractions showed no differences. To visualize the subcellular expression of CTSB in the pancreas we labeled paraffin-embedded unstimulated pancreatic tissues with a CTSB-specific (red) and a GM130-specific (green) antibody followed by confocal microscopy (Fig. 3a). GM130 served as a Golgi marker. A co-localization of both proteins was found in both CLN8^−/−^ and wild type mice indicating the presence of lysosomal enzymes in the Golgi despite absence of CLN8, albeit to a lesser extent in the knockouts. Labeling of CTSB (red) and the lysosomal marker LAMP-2 (green) indicated that CTSB was still localized in the lysosomes of CLN8^−/−^ mice (Fig. 3b). Labeling of CTSB (red) and the zymogen granule marker syncollin (cyan) also demonstrated co-localization under control conditions (0 h) in the pancreas that was equal in both groups. Induction of acute pancreatitis increased CTSB/Syncollin colocalization which was less pronounced in CLN8^−/−^ (Fig. 3c). Taken together, absence of CLN8 in the pancreas significantly reduced the amount of CTSB in the Golgi system, but not in the lysosome or the zymogen granule. CTSB can therefore co-localize with trypsinogen and cause initiation of acute pancreatitis, even though to a lesser extent in the CLN8^−/−^ mice. These findings suggest the presence of alternative intracellular transport pathways in the pancreas which allow the CTSB transfer from the ER to the lysosomal and secretory compartment, respectively.

### Loss of CLN8 alters mitochondrial morphology and function

**Fig. 4:**
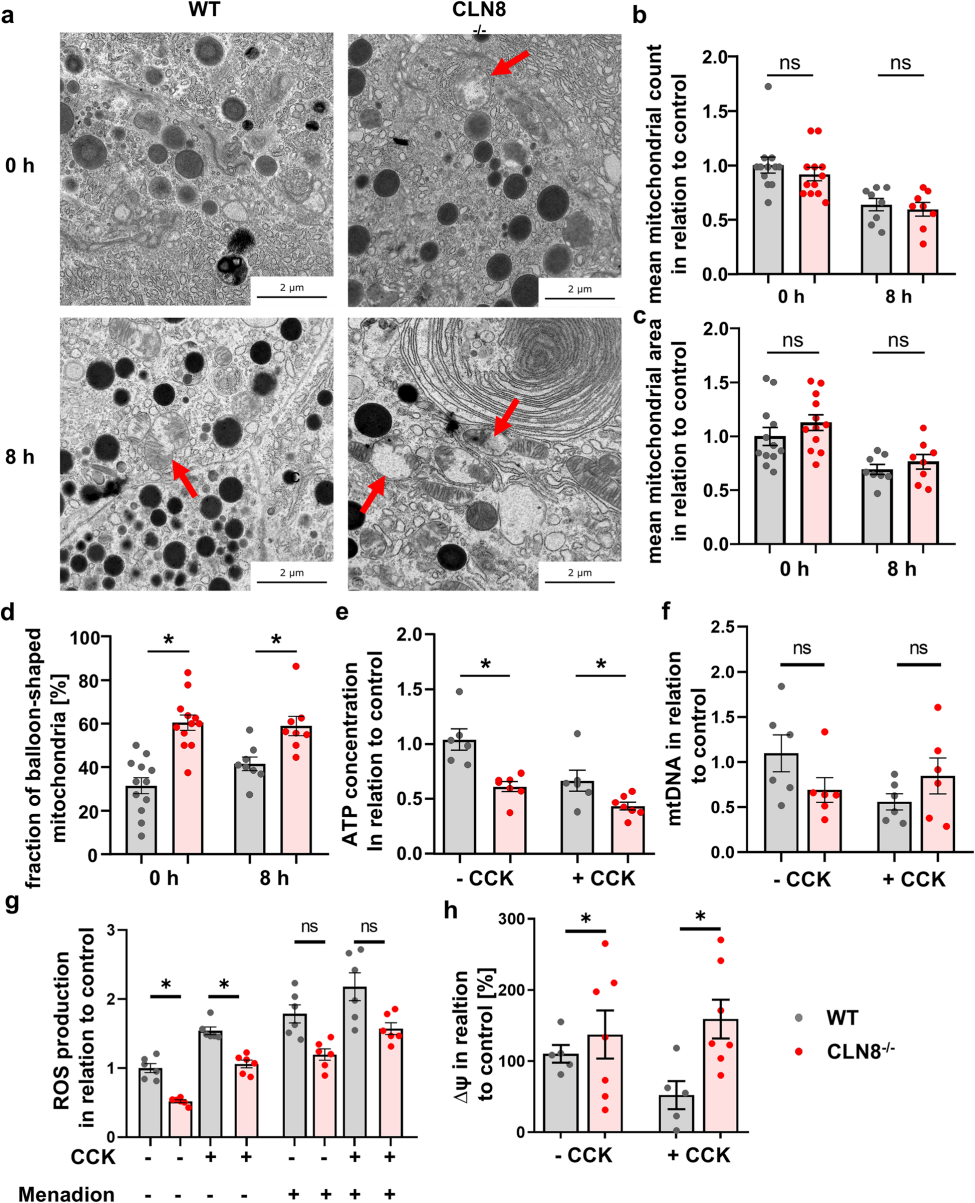
Analysis of mitochondria morphology and metabolism in the pancreas of CLN8^−/−^ and wild type mice. **a** Electron microscopic images of pancreatic acinar cells of wild type mice (WT) and CLN8-deficient mice (CLN8^−/−^) at physiological conditions (0 h) and the late phase of the disease (8 h) displayed a higher number of ballooned shape mitochondria (red arrows) in CLN8 knockout mice at 0 h and 8 h of caerulein-induced pancreatitis. In wild type mice, morphological alterations were less distinct. **b**, **c** Quantification of the number and area of mitochondria in electron microscopic images showed no differences between CLN8^−/−^ and wild type pancreas. **d** The proportion of balloon-shaped mitochondria out of the total amount of mitochondria significantly increased in the CLN8-deficient pancreas compared to controls. **e** Under both unstimulated (- CCK) and CCK-stimulated (+ CCK) conditions ATP concentrations were significantly lower in homogenates of CLN8 knockout acinar cells compared to homogenates of wild type acinar cells. **f** The mitochondrial DNA (mtDNA) amount showed no differences in WT and CLN8-deficient acinar cells irrespective of cholecystokinin (CCK) stimulation. **g** The ROS production was significantly reduced in CLN8-deficient acinar cells before and after CCK-stimulation. Usage of menadione as a positive control diminished this effect. **h** The maximal mitochondrial membrane potential (∆ψ) of CLN8-deficient acinar cells was significantly enhanced under physiological conditions and after CCK stimulation compared to WT acinar cells. For the electron microscopic imaging and quantification, three animals per strain and per time point and 4 visual fields of each were analyzed. For the acinar cell experiments, at least 6 animals were used. The measurements were performed in duplicates. Values are mean ± SEM. * denotes p < 0.05

As we observed a deteriorated course of acute pancreatitis in the CLN8-knockouts we then focused on subcellular acinar morphology as well as mitochondrial function and ER. Electron microscopy imaging of CLN8-deficient pancreas showed an increased number of vesicles resembling autophagosomes, dysmorphic mitochondria, and an ER with swollen appearance. These alterations were more pronounced at 8 h of pancreatitis (Fig. 4a). Analysis of the total mitochondrial number and covered area by mitochondria showed no difference between CLN8-deficient and wild type mice, but a reduction during acute pancreatitis (8 h) (Fig. 4b, c). The fraction of balloon-shaped mitochondria was already significantly increased in the CLN8-deficient pancreas under unstimulated conditions and remained elevated during acute pancreatitis (Fig. 4d).

Mitochondrial function was determined in isolated acinar cells from CLN8-competent and –deficient mice after supramaximal CCK stimulation followed by measurement of the ATP concentration (Fig. 4e). The ATP concentration was significantly lower in homogenates of CLN8^−/−^ acinar cells under unstimulated conditions and further decreased after CCK-stimulation. Mitochondrial DNA (mtDNA) was extracted from isolated acinar cells and quantified by qPCR to estimate mitochondria amount. The fraction of mtDNA was similar between WT and CLN8^−/−^ acinar cells and independent of CCK-stimulation (Fig. 4f). Measurement of reactive oxygen species (ROS) showed a significantly reduced ROS-production in CLN8-deficient acinar cells in contrast to wildtypes. CCK-stimulation augmented the intracellular ROS production in both WT and CLN8^−/−^ acinar cells. Additional stimulation of acinar cells with menadione serving as a positive control [19] increased ROS production in both CLN8-competent and CLN8-deficient cells but no significant differences between the groups were detected (Fig. 4g). We also measured the maximal mitochondrial membrane potential (∆ψ) in isolated acinar cells prior and after CCK-stimulation. Depletion of CLN8 increased ∆ψ, which did not decline after CCK-stimulation like in WT cells (Fig. 4h). Taken together, there is strong evidence that deficiency of CLN8 affects both mitochondrial shape and function.

### ER-stress is elevated in absence of CLN8

**Fig. 5:**
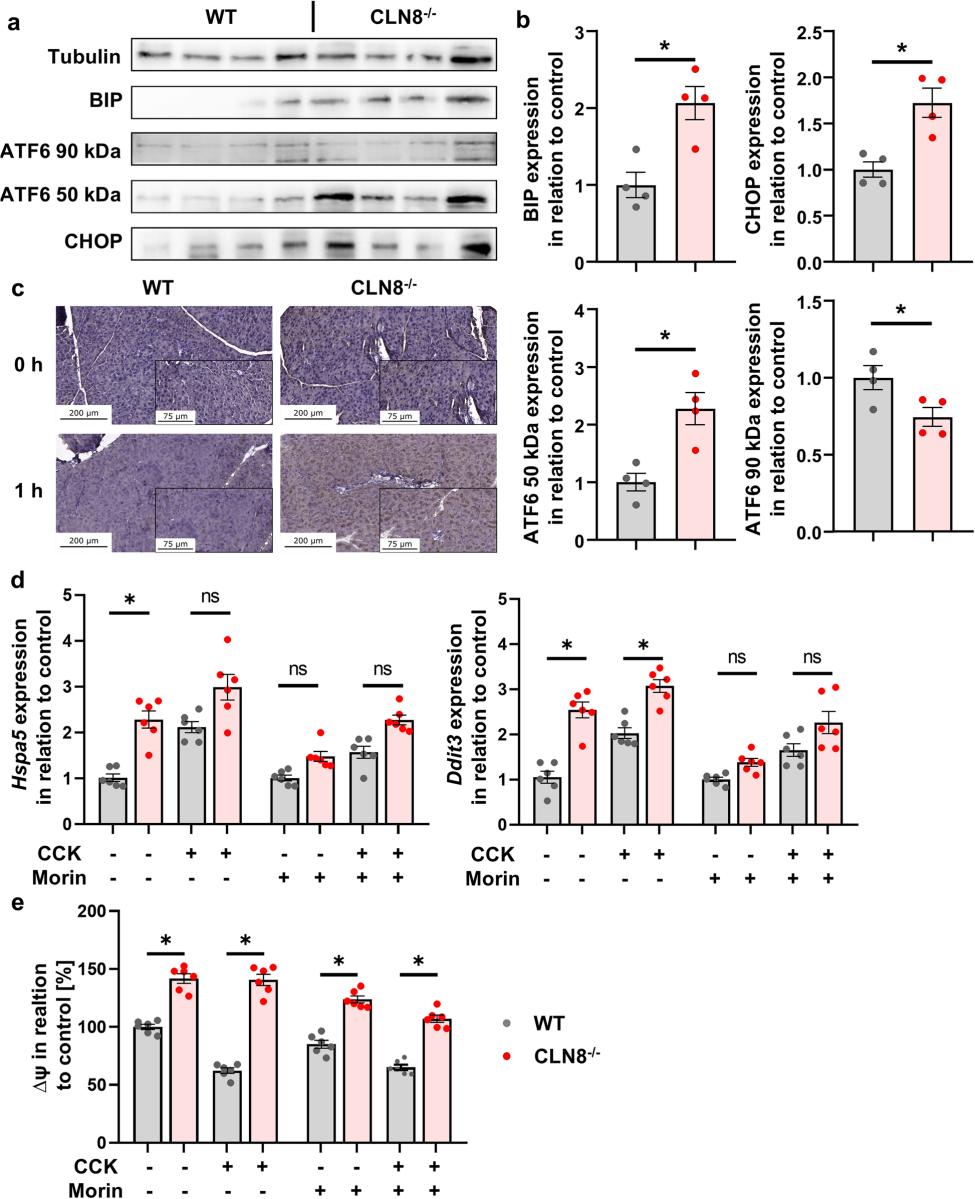
ER-stress is dependent on CLN8. **a**, **b** Immunoblot analysis and densitometric quantification of ER stress markers BIP, CHOP, and ATF6 in pancreas homogenates of unstimulated mice indicated an increase of ER stress in the absence of CLN8. **c** Immunohistochemical stainings of paraffin-embedded pancreatic tissues confirmed the increase in BIP in CLN8-deficient mice at 0 h and 1 h of caerulein pancreatitis. **d** The qPCR analysis showed a significant upregulation of mRNA expression of both *Hspa5* and *Ddit3* in CLN8^−/−^ acinar cells compared to WT cells. CCK stimulation further upregulated their expression. Incubation of acinar cells with the ER-stress inhibitor morin diminished the differences in gene expression between CLN8-deficient and WT acinar cells. **e** In isolated acinar cells, morin did not affect the mitochondrial membrane potential as ∆ψ remained significantly elevated in CLN8-deficient cells compared to WT. For immunoblot analysis four animals were used and qPCR analysis was done from six animals per strain and condition. The measurements were performed in duplicates. Values are mean ± SEM. * denotes p < 0.05

Besides abnormal ER-morphology, ER stress was elevated in CLN8-deficient mice as demonstrated by an enhanced expression of the stress-markers BIP, CHOP, and ATF6 in pancreas homogenates (Fig. 5a, b).

Immunohistochemical labeling of paraffin-embedded pancreatic tissues with a BIP-specific antibody showed a higher expression in CLN8^−/−^ mice compared to wild types under both physiological conditions and after onset of pancreatitis (Fig. 5c). Measurement of mRNA expression of the ER-stress markers *Hspa5* and *Ddit3*, which encode for BIP and CHOP, respectively, yielded similar results. CCK-stimulation upregulated the mRNA expression of these ER-stress markers. On the other hand, treatment with morin [20], an ER-stress inhibitor, significantly reduced the gene expression of *Hspa5* and *Ddit3* in both WT and CLN8^−/−^ cells and the differences between both strains were no longer significant (Fig. 5d). To evaluate the effect of ER-stress inhibition on mitochondrial function, the maximal mitochondrial membrane potential (ΔΨ) was determined in morin-stimulated acinar cells (Fig. 5e). This parameter turned out to be independent of ER-inhibition, as we did not observe a significant change after morin treatment. Taken together, loss of CLN8 in pancreatic acinar cells is linked to an increased ER-stress. Inhibition of ER-stress does not alter mitochondrial function.

### Loss of CLN8 induces autophagy in acini

**Fig. 6:**
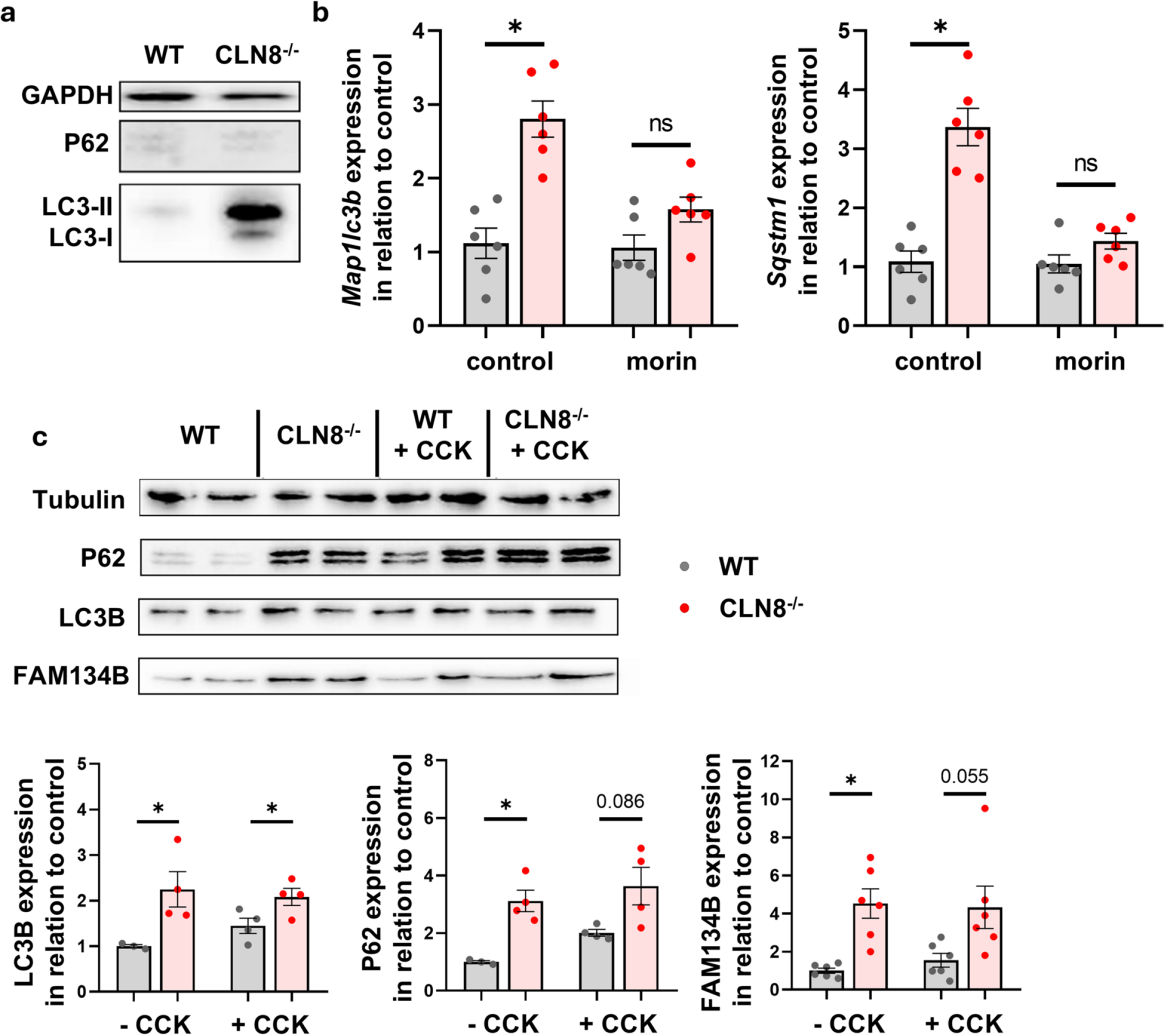
Analysis of the autophagic flux in CLN8^−/−^ and wild type acinar cells. **a** Expression of the autophagy marker LC3B was strongly increased in CLN8-deficient pancreas (CLN8^−/−^) compared to wild type (WT), whereas P62 expression remained unaffected as demonstrated by immunoblot analysis of pancreas homogenates. **b** Gene expression analysis by qPCR showed significant upregulation of both *Map1lc3b* and *Sqstm1* gene expression in the CLN8-deficient acinar cells compared to WT. Stimulation with morin downregulated the gene expression of both mRNAs and the differences between WT and CLN8.^−/−^ were not significant anymore. **c** Immunoblot analysis and densitometric quantification for LC3B, P62, and FAM134B protein expression in acinar cell homogenates after incubation with E-64-d. Protein expression was significantly enhanced in CLN8-deficient acinar cells compared to WT under normal conditions. Additional CCK-stimulation increased the protein expression primarily in the WT cells. Densitometric quantification was performed with four (LC3B, P62) or six (FAM134B) biological replicates. For the qPCR, six biological replicates were used and measurements were performed in triplicates. Values are mean ± SEM. * denotes p < 0.05

**Fig. 7:**
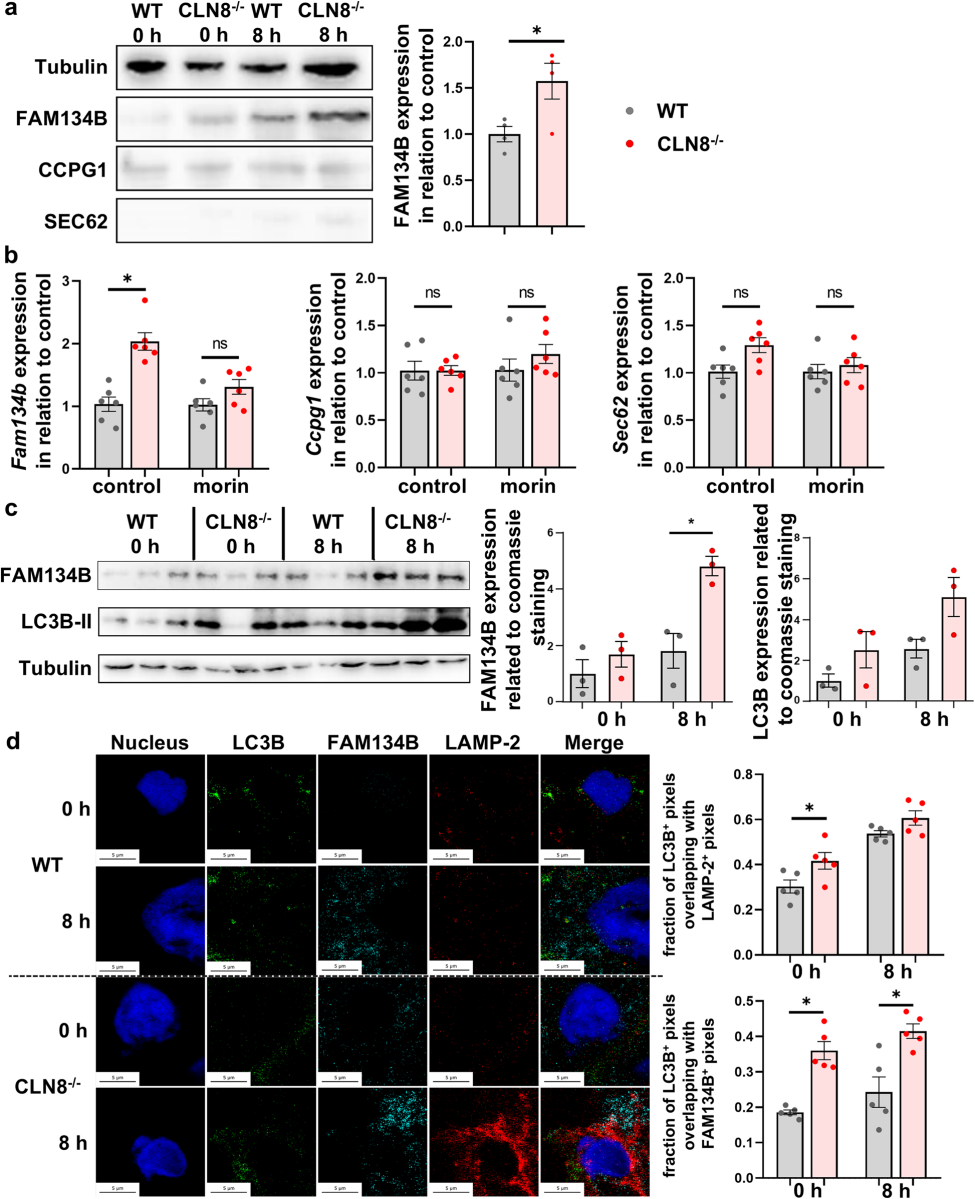
Evaluation of ER-phagy in the pancreas of CLN8-knockout mice and controls. **a** Expression of the ER-phagy receptor FAM134B was upregulated in CLN8^−/−^ pancreas under physiological conditions (0 h) compared to wild types, which further increased upon caerulein-induction (8 h). The expression of the ER-phagy receptors CCPG1 and SEC62 remained at low levels in CLN8 mice and during acute pancreatitis. Densitometric quantification of the FAM134b bands at unstimulated conditions (0 h) underlined its higher expression in the pancreas of CLN8-deficient mice. **b** Gene expression analysis by qPCR also showed a significant upregulation of *Fam134b* mRNA in CLN8-deficient acinar cells. Morin reduced *Fam134b* expression, so that the differences between WT and CLN8.^−/−^ were not significant anymore. Gene expression of *Ccpg1* and *Sec62* showed comparable results between WT and CLN8-deficient acinar cells and after morin treatment. **c** Co-precipitation using a LC3B-specific antibody followed by immunoblot and densitometric analysis of pancreas homogenates demonstrated an increased interaction of LC3B-II with FAM134B in CLN8-deficient mice at control conditions (0 h) which markedly increased during acute pancreatitis (8 h). Coomassie staining was used for normalization. Tubulin expression of the input material was used as loading control. **d** Labeling of LC3B (green) and LAMP-2 (red) in paraffin-embedded pancreatic tissues demonstrated an increased co-localization in absence of CLN8 indicating enhanced autophagolysosome formation at basal condition (0 h). This difference diminished at 8 h of acute pancreatitis. Analysis of LC3B (green) and FAM134B (cyan) pixels reflected a higher co-localization both at 0 h (control) and 8 h acute pancreatitis in the knockouts. Immunoblot analysis was performed with 4 (**a**) or 3 (**c**) biological replicates. For the acinar cell experiments, six animals of each strain were used. Visual fields of 5 animals were used for colocalization analysis. Values are mean ± SEM. * denotes p < 0.05

Given the fact that cellular stress causes cellular protein degradation in many organ systems [21, 22] we investigated autophagy in the pancreas and monitored the expression of the autophagy marker proteins LC3B and P62 (Fig. 6a). Immunoblot analysis of pancreatic homogenates showed a strong increase of LC3B-I and LC3B-II bands in CLN8-deficient mice while the P62 signal was unaltered. In addition, we found a significant increase of *Map1lc3b* and *Sqstm1* mRNA expression in CLN8-deficient acinar cells (Fig. 6b). Interestingly, inhibition of ER-stress by morin downregulated the gene expression of both autophagy markers and differences between WT and CLN8^−/−^ disappeared. Similar results could be obtained for the autophagy-related genes *Atg3, Atg5, Atg7, Atg10, Becn1,* and *Becn2* as well as the mitophagy-related genes *Pink1* and *Prkn* (Fig. S3). Inhibition of the autophagic flux by the cysteine protease inhibitor E-64-d caused a significant increase in LC3B, P62, and the ER-phagy receptor FAM134B protein expression in CLN8^−/−^ acinar cells compared to wild types as reflected by densitometric analysis of immunoblots (Fig. 6c). CCK-stimulation further elevated protein expression. As ER stress can cause autophagy of ER-components we next investigated the expression of different ER-phagy receptors and their possible interaction with LC3B. Before induction of pancreatitis FAM134B expression was higher in CLN8-deficient mice and augmented after 8 h, as confirmed by densitometric analysis, whereas the expression of CCPG1 seemed to be independent of CLN8. Bands of the ER-phagy receptor SEC62 were hardly detectable and not inducible by caerulein-stimulation (Fig. 7a). In addition, qPCR analysis showed significant upregulation of *Fam134b* mRNA in CLN8-deficient acinar cells, but not of *Ccpg1* and *Sec62* (Fig. 7b). Inhibition of ER-stress by morin diminished the difference in *Fam134b* gene expression between CLN8^−/−^ and wild type cells. Evidence of ER-phagy was visualized by a direct interaction of LC3B and FAM134B in pancreas homogenates after co-immunoprecipitation. The proteins were first precipitated by a specific antibody for LC3B followed by immunoblotting of the precipitates with FAM134B- and LC3B-specific antibodies. The densities of both proteins were quantified separately and normalized to coomassie staining of the SDS-PAGE gels. Tubulin expression of the input material was used as loading control. Loss of CLN8 was associated with a stronger interaction between both LC3B and FAM134B that further increased at 8 h of acute pancreatitis (Fig. 7c). The higher amount of FAM134B pulled down by the co-immunoprecipitation might be the consequence of the higher LC3B expression in the CLN8-deficient mice. On the other side, wildtype mice showed a much weaker LC3B band and hardly any FAM134B signal at 0 h. While protein expression also increased during acute pancreatitis it remained significantly weaker compared to the knockouts. Immunofluorescence labeling of LC3B (green), FAM134B (cyan) and LAMP-2 (red) in pancreatic tissues at 0 h and 8 h of acute pancreatitis visualized a higher co-localization of LC3B with both FAM134B and LAMP-2 under control conditions (0 h) in CLN8-deficient mice (Fig. 7d). During acute pancreatitis (8 h) co-localization of LC3B and LAMP-2 increased and results were similar between wild type and CLN8^−/−^ mice. In contrast, co-localization of LC3B with FAM134B was comparable between 0 h and 8 h and more intense in the CLN8-deficient mice. Our results demonstrate that ER-phagy, as a part of autophagy, rises in absence of CLN8 and during acute pancreatitis. Overall autophagolysosome formation seems to be independent of CLN8 in acute pancreatitis, as co-localization of LC3B with LAMP-2 was not elevated in CLN8-deficient mice.

### CLN8-deficient 266–6 cells show elevated ER-stress and ER-phagy

**Fig. 8:**
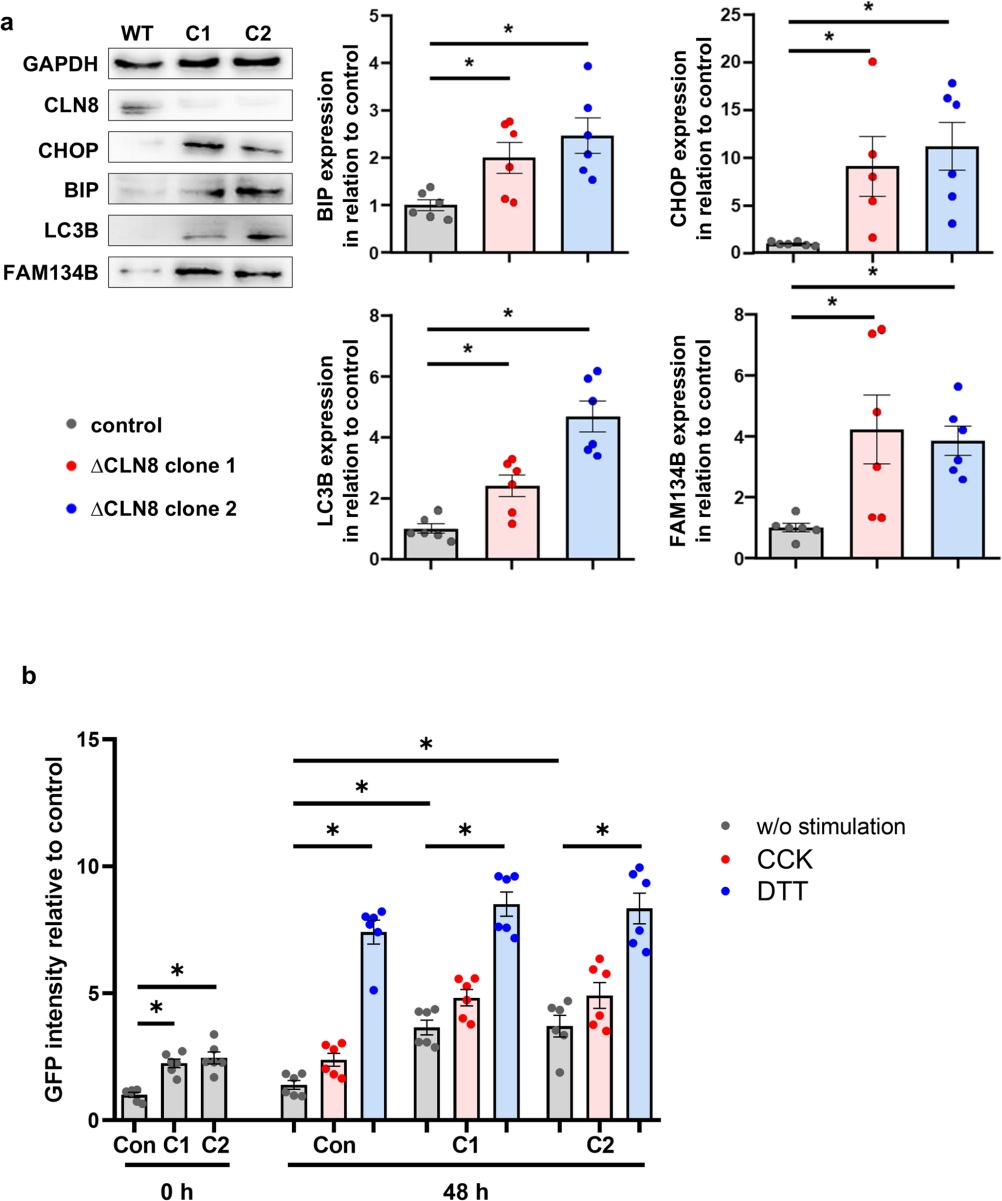
266–6 ΔCLN8 cells are characterized by similar cellular responses upon inactivation of CLN8. **a** Immunoblot analysis followed by densitometric quantification of cell lysates confirmed the knockout of CLN8 in two CLN8-deficient 266–6 cell clones (∆CLN8 clone 1, ∆CLN8 clone 2) paralleled by a significantly elevated expression of the ER stress markers BIP and CHOP, the autophagy marker LC3B, and the ER-phagy receptor FAM134B compared to controls. **b** Measurement of GFP expression after transfection of clones 1 (C1) and 2 (C2) and controls (Con) with a GFP-ER stress-reporter plasmid following stimulation with either CCK or DTT as a positive control was used for assessment of ER-stress. Equal numbers of cells were seeded. Both CCK and DTT increased ER-stress after 48 h and increments were stronger in CLN8-deficient cells. The values represent the results of six independent experiments and the measurements were performed in duplicates. Values are mean ± SEM. * denotes p < 0.05

The 266–6 cell line is derived from a pancreatic acinar cell tumor with preserved acinar cell morphology. Inactivation of CLN8 expression in 266–6 cells by CRISPR/Cas9 genome editing led to two cell clones, which harbored a homozygote frameshift mutation in exon 2 of the *Cln8* gene, resulting in the blockade of synthesis of a functional protein CLN8. Absence of CLN8 was confirmed on protein level by Western Blot analysis of cell lysates from both cell clones (Fig. 8a). Inactivation of CLN8 resulted in a more three-dimensional cluster-like culture phenotype (Fig. S4a). CLN8-deficient 266–6 cells also showed a significantly elevated expression of the ER-stress marker proteins BIP and CHOP as well as of the autophagy marker LC3B and the ER-phagy receptor FAM134B compared to control cells (Fig. 8a). These findings were supported by mRNA overexpression of *Map1lc3b, Sqstm1, Hspa5, Ddit3*, and *Fam134b* in the CLN8-deficient cells (Fig. S4c). Quantification of cell proliferation was conducted using the cell counting kit-8 (CCK-8) and proliferation was monitored for up to 72 h. While unstimulated CLN8-expressing 266–6 cells showed a nearly fourfold increase in cell numbers, supramaximal CCK-concentrations markedly diminished cell proliferation. Unlike controls, CLN8-deficient clones hardly proliferated at all and addition of CCK had no effect (Fig. S4b). In an alternative approach for ER-stress quantification, cells were transfected with an ER-stress reporter plasmid encoding the human gene *XbpI* fused to the *Gfp* gene. ER stress results in alternative splicing of the XBPI transcript and higher expression of GFP that is detectable by fluorescence measurements. Under resting conditions, CLN8-deficient 266–6 cells showed higher GFP intensities, reflecting elevated ER stress levels. Stimulation of CLN8-competent control cells and CLN8-deficient cell clones either with dithiothreitol (DTT), a known ER stress inducer used as a positive control, or with supramaximal CCK further increased GFP intensities in all three groups (Fig. 8b). ER-stress after CCK stimulation was also higher in both CLN8-deficient 266–6 clones compared to controls.

Taken together, deficiency of CLN8 in the pancreatic acinar tumor cell line 266–6 resulted in a disturbed cell colony morphology and a similar cell physiology like pancreatic acinar cells from CLN8^−/−^ mice as shown by elevated expression of the autophagy marker LC3B, the ER-phagy receptor FAM134B, the ER-stress proteins BIP and CHOP as well as the ER-stress induction after CCK-stimulation.

### CLN8 expression in human pancreas

We checked public resources for CLN8 expression in the human pancreas and sequenced blood samples from healthy individuals and patients with acute recurrent or chronic pancreatitis for *Cln8* gene variants. Moreover, we stained human chronic pancreatitis tissue samples for CLN8 and the ER-stress protein CHOP. According to databases CLN8 is expressed in the human pancreas at least on mRNA level (Expression Atlas: ensg00000182372; GTEX Portal: ENSG00000182372.10; The Human Protein Atlas: ENSG00000182372-CLN8). Immunostaining of CLN8 (red) and CHOP (cyan) of pancreas tissues from patients with chronic pancreatitis confirmed localization of CLN8 in human pancreatic acinar cells and indicated presence of ER-stress in chronically inflamed pancreas (Fig. S5a). The possible interaction of ER-stress and CLN8 in human pancreatitis and implications for the clinical course warrant deeper analysis. Whole genome sequencing of the *Cln8* gene in individuals from two populations-based cohorts, that originated from the “Study of Health in Pomerania” (SHIP cohort 1 and 2) [23], and patients with alcoholic chronic pancreatitis (ACP), idiopathic chronic pancreatitis (ICP) and recurrent acute pancreatitis (RAP), respectively, identified three heterozygous single nucleotide polymorphisms (SNPs) in healthy individuals but no variant in the pancreatitis cohort (Fig. S5b). Two of the identified SNPs were silent mutations (p.Thr111 =; p.Val236 =) and the third one was a missense mutation, resulting in the exchange of asparagine to serine (p.Asn125Ser). The effects of all three variants on the protein function were assessed by PolyPhen-2 [24] and predicted as “benign”.

## Discussion

The early events of acute pancreatitis are believed to start inside acinar cells and are characterized by a premature and intracellular activation of digestive proteases starting with trypsinogen. 

Cathepsin B is a trypsinogen activator and is synthesized as a pro-enzyme in the rough ER before it is transported to the trans-Golgi system by the CLN8-CLN6-dependent EGRESS complex and the coat protein complex II (COPII), where pro-CTSB undergoes posttranslational modifications and sorting [11, 12]. Besides its main localization in the lysosome a small amount of CTSB is found in the secretory compartment, where it is able to co-localize with trypsinogen [4, 25, 26].

In our study we could demonstrate that loss of CLN8 did not prevent from onset of acute pancreatitis although active CTSB expression was markedly reduced in the secretory compartment, the site where initial trypsinogen activation occurs. Cathepsins were localized in the Golgi to a lesser extent but were still detectable in their final compartments, the lysosomes and zymogen granules. Previous studies postulated a disrupted trafficking of lysosomal enzymes followed by their absence in the lysosomal compartment in CLN8-depleted mice [11, 12]. Our findings suggest the existence of either an alternative, canonical transport mechanism or the presence of non-canonical pathways by which cathepsins can exit the endoplasmic reticulum to reach the lysosome and zymogen granules despite absence of CLN8.

Autophagy is an essential process responsible for the maintenance of cellular homeostasis as it mediates degradation of damaged proteins and thus improves the survival of cells. Both, impaired autophagy and autophagic flux can occur during acute pancreatitis [7, 8, 27, 28]. CLN8-deficient pancreas exhibited a dramatic increase in autophagy shown by LC3B expression paralleled by a formation of large vacuoles resembling autophagosomes or autophagolysosomes. A CLN8-related effect on autophagy was already investigated in a CLN8-deficient zebrafish model that showed impaired autophagy with increased expression of LC3 and the late endosome marker Rab7 [29]. We suspect the formation of autophagolysosomes as indicated by a higher expression of the ER-phagy receptor FAM134B along with LC3B/FAM134B and LAMP-2/LC3B co-localization in CLN8^−/−^ pancreatic tissues. Induction of acute pancreatitis strongly increased FAM134B bound to LC3B and FAM134B/LC3B co-localization underlining that ER-phagy may contribute to disease development in CLN8 knockout mice. The direct interaction of FAM134B with LC3B denotes autophagosome constitution containing ER material and pro-forms of cathepsins [30]. However, the expression of the ER-phagy receptors CCPG1 and SEC62 was independent of CLN8 and onset of acute pancreatitis. While CCPG1 is an important regulator of pancreatic protein homeostasis [31] and despite its known function for recovery from ER-stress and transport of ER-components to autolysosomes [32], the role of SEC62 has not been investigated in the pancreas yet. For a more meticulous analysis of ER-phagy receptors in acute pancreatitis, more studies are necessary.

In the early disease phase total activity of CTSB as well as trypsin were lower in CLN8^−/−^ acinar cells and mice, which is most likely attributed to the reduced overall enzyme activation in the secretory compartment. There are several underlying reasons such as a suboptimal pH milieu in organelles including the zymogen granules. The expression of the lysosomal H^+^/ATPase is cell type-dependent and significantly reduced in CLN8-deficient mice [15], which leads to an elevated lysosomal pH in CLN8^−/−^ tissues [13, 33] and to an inadequate catalytic activity of cysteine proteinases such as CTSB [34, 35]. Absence of CLN8 might also hamper organelle co-localization, as CLN8 participates in vesicular distribution and its loss is linked to lysosomal malfunction [13, 36]. However, recent studies questioned the importance of CTSB originating from lysosomes for disease initiation because zymogen granules already contain small amounts of CTSB. Onset of pancreatitis is not prevented by lysosomal permeabilization so that lysosome-derived CTSB and its transport into the secretory compartment seems to be expendable for acute pancreatitis [4, 5]. Furthermore, a recent study could show that mitochondrial damage induced by high concentrations of L-lysine is also able to induce trypsin activation and necrosis in isolated acinar cells of rodents [37].

ER-stress impairs the acinar cell function, causes the formation of dysfunctional mitochondria [8] and contributes to acute pancreatitis [10, 38–41]. There is a relation between ER-stress response and CLN8 expression, which is not surprising as CLN8 is an ER-membrane associated protein mediating the export of proteins from the ER. However, the overexpression of ER-stress marker proteins ATF6, BIP, and CHOP in CLN8^−/−^ mice did not cause spontaneous acute pancreatitis so that its initiation still requires an exogenous stimulus. The observed CLN8-dependent modulation of ER-stress is in line with previous studies that mentioned an elevation of ER stress-related molecules and activation of ER stress pathways in central nervous system structures of *Cln8* mutated mice with motor neuron degeneration (mnd) [14]. Whether ER stress is the immediate cause for disease onset or simply a side effect of an acute inflammatory condition cannot be answered by our results. Incubation of isolated acinar cells with the ER-stress inhibitor morin [20] downregulated the expression of ER-stress markers as well as autophagy-related proteins but did not change the abnormal mitochondrial membrane potential in isolated CLN8^−/−^ acinar cells. Therefore, ER-stress seems to be closely linked to autophagy and an inducer for ER-phagy in acinar cells. 

Morphological and functional defects of mitochondria can result from ER-stress. We confirmed the presence of dysmorphic mitochondria and an impaired mitochondrial metabolism by reduced ATP concentrations as well as a significant reduction in ROS production and a disrupted mitochondrial membrane potential in CLN8-knockout mice. Reduced mitochondrial metabolism in turn can contribute to a decline of protease activation in CLN8^−/−^ acinar cells. Whether this decline is also related to a defective clearance of intracellular Ca^2+^ loads and an impaired mitochondrial Ca^2+^ uptake needs further evaluation. A sustained elevation of intracellular Ca^2+^ levels in acini typically occurs after CCK stimulation and triggers acute pancreatitis [42]. In the late stages of acute pancreatitis, disease severity was aggravated in CLN8^−/−^ mice as documented by increased levels of serum enzymes, trypsin activity, and higher local damage. The more severe course may eventually results from ER-stress and mitochondrial dysfunction that started in the early phase and would be in line with results from previous studies highlighting the association of ER stress [10, 41, 43, 44] and mitochondrial damage [8, 9, 45, 46] with injury in acute pancreatitis.

Our study contains limitations. Acute pancreatitis was only examined in one in vivo model which was caused by the secretagogue caerulein. Furthermore, we only used a mouse strain with a global CLN8-deficiency and an acinar cell-specific knockout of CLN8 using the Cre/loxP system could expand the knowledge on acinar cell specific effects of CLN8. As CLN6 binds to CLN8 to form the EGRESS-complex but does not travel from the ER to the Golgi along with CLN8 [12] the investigation of other ceroid lipofuscinosis (CLN) proteins in acute pancreatitis would be helpful. Finally, the connection between ER-stress, autophagy, mitochondrial function, and protease activity, which all act on disease severity, was only analyzed in vitro by inhibitors. To obtain a complete overview, in vivo experiments will be necessary.

In summary, we provide evidence that acute pancreatitis is inducible despite absence of the ER-cargo protein CLN8 whose original function is to ensure the correct intracellular enzyme transportation. The detection of cathepsins in compartments other than the ER suggests the presence of alternative enzyme delivery routes apart from the EGRESS complex. Secondly, ER-stress, autophagy, and mitochondrial dysfunction due to loss of CLN8 may contribute to pancreatic injury at later disease stages.

## Methods

### In vivo mouse model

Animal experiments were performed according to the German law and after approval by the institutional animal care facility (Landesamt für Landwirtschaft, Lebensmittelsicherheit und Fischerei Mecklenburg-Vorpommern (LALLF-MV)). Wild type C57BL/6 J mice (RRID: IMSR_JAX:000664) were obtained by Charles River Laboratories (Sulzfeld, Germany) and CLN8-deficient B6.KB2-*Cln8*^*mnd*^/MsrJ mice (CLN8^−/−^, RRID:IMSR_JAX:001612) by The Jackson Laboratories (Bar Harbor, USA). Acute pancreatitis was induced in 8–12 weeks-old C57BL/6 J and CLN8^−/−^ mice by hourly intraperitoneal caerulein injections (50 µg/kg body weight) for up to 8 h. Mice were sacrificed one hour after the last caerulein application. The blood was sampled and the organs were asservated for further analysis. Therefore, one part of the organ was fixed in 4.5% paraformaldehyde for paraffin-embedding, one part was embedded in TissueTec O.C.T. compound (Sakura Finetek, Alphen aan den Rijn, The Netherlands) and one part was snap frozen in liquid nitrogen and stored afterward in −80 °C.

Biochemical analysis were performed with pancreas homogenates. Therefore, the pancreas was homogenized by using a dounce homogenizer. The organ lysate was sonicated two times for 10 s, centrifuged at 20,800 g for 10 min at 4 °C and the supernatant containing the proteins was stored at −80 °C for further experiments.

### Acinar cell isolation and stimulation

Acinar cells from C57BL/6J and CLN8-deficient mice were isolated as described previously [47]. Cultivation of cells was performed in Dulbecco’s modified Eagle medium, supplemented with 10 mM HEPES and 2% BSA. Cells were stimulated with 1 µM CCK. Measurement of cellular necrosis and protease activation was performed before and 20, 40, and 60 min after CCK-stimulation, with untreated cells serving as controls, as described previously [5]. Cells were stimulated with morin (100 µM) for 60 min or E-64-d (50 µM) for 30 min in prior to CCK-stimulation.

### Mitochondrial membrane potential measurement

Freshly isolated acinar cells were stimulated with 1 µM CCK, with untreated cells serving as controls. After 30 min stimulation, acinar cells were washed and loaded with tetramethylrhodamine methyl ester (TMRM; 1 µM) and incubated for 20 min at 37 °C, followed by fluorescence intensity measurement. The mitochondrial membrane is depolarized by carbonyl cyanide 3-chlorophenylhydrazone (CCCP; 15 µM) stimulation and fluorescence intensity was measured again. Normalization was performed against DNA measurement by propidium iodide.

### ROS measurement

Isolated acinar cells were loaded with 5 µM CellROX Deep Red for 30 min. Afterwards, cells washed and stimulated with 1 µM CCK for 20 min in acinar cell media at 37 °C. After incubation, fluorescence intensity was measured. Menadione treatment of the acinar cells served as positive control. Normalization was performed against DNA measurement by propidium iodide.

### Subcellular fraction

Mice were sacrificed and the pancreas was homogenized using a dounce homogenizer and a homogenization buffer containing 240 mM sucrose, 5 mM MOPS, and 1 mM magnesium sulfate with a pH of 6.5. Several subcellular fractions of the pancreas were obtained by differential centrifugation. After centrifugation at 150 g at 4 °C for 10 min, the perinuclear fraction was obtained. The samples were centrifuged at 470 g for 15 min at 4 °C to obtain the zymogen granule-enriched fraction, at 12,200 g for 12 min at 4 °C for the lysosome-enriched fraction, and at 20,800 g for 12 min at 4 °C for the cytosolic fraction. After each centrifugation step, the pellet contains the aimed fraction and the supernatant was used for the next centrifugation step. The pellets were suspended in PBS, sonicated two times for 10 s, snap-frozen in liquid nitrogen, and stored at −80 °C.

### Biochemical assays

Amylase and lipase activity measurements were performed with serum or pancreas homogenate samples using the photometric Amyl2 (03183742122) and LipC (03029590322) kit from Roche. Measurement was done as kinetics over 30 min at 37 °C.

Protease activity was measured using specific fluorometric substrates and protease-specific buffers. Measurement of CTSB and CTSL was done in a buffer containing 100 mM sodium acetate, 5 mM calcium chloride, 10 mM DTT, and a pH of 5.5 or 4, respectively, after adding 10 mM AMC-Arg2 for CTSB and AMC-Phe-Arg for CTSL. The activity of trypsin was assessed by using 10 mM R110-Ile-Pro-Arg for protease activity in living cells or Boc-Gln-Ala-Arg-AMC in cell homogenates and a buffer containing 100 mM Tris and 5 mM calcium chloride with a pH at 8. To determine total trypsinogen content, samples were preincubated with enterokinase (10 mU) for 30 min at 37 °C before trypsin activity measurement. Normalization of activity was done by protein concentration, which was assessed by a Bradford assay.

### Histology

Histological analysis of organs was done by staining of paraffin-embedded tissue slides with hematoxylin and eosin (HE). Scoring of the tissue damage was performed using a modified histoscore adapted from Niederau et al*.* [48]. Immunological labeling of pancreatic tissue was performed with paraffin- and cryo-embedded slides. Before staining, antigen retrieval of paraffin-embedded slides was performed using the antigen retrieval solution of DAKO (Carpinteria, USA). Blocking of the slides was done with PBS containing 20% BSA and 0.01% Triton X-100. Primary antibodies were diluted in the blocking buffer. Development of the slides for immunohistochemical staining was carried out by using the DAB substrate kit from Vector Laboratories (Newark, USA).

Microscopy imaging was done using an Olympus FLUOVIEW FV100 fluorescence microscope (Olympus, Hamburg, Germany), a Pannoramic Midi (Sysmex, Norderstedt, Germany), and a confocal microscope Leica Stellaris 8 (Leica, Wetzlar, Germany). Analysis of co-localization of two signals was performed using the software Fiji [49] with the plugin JACoB [50].

### Electron microscopy

Tissue was sliced into small pieces and incubated in 2% OsO_4_ and 0.1 M Cacodylatbuffer pH 7.4 for 2 h on ice. Afterwards, samples were washed three times with 0.1 M Cacodylatbuffer pH 7.4 and stored on ice for further procedure. The osmium-fixed tissue pieces were embedded in Glycidether 100 (Serva, Heidelberg, Germany), cut with diamond knives (Science Service GmbH, Munich, Germany) with a Leica ultratome (Leica EM UC7, Leica Biosystems, Wetzlar, Germany) to 500 and 750 nm thick semi-thin slides. While, the 70–90 nm ultrathin sections were stained with two-step staining procedure consist of uranyl acetate (Merck, Darmstadt, Germany) and lead citrate (Sigma-Aldrich, Darmstadt, Germany) as the standard routine contrasting technique for electron microscopy and examined with Libra 120 electron microscope (Carl Zeiss, Jena, Germany).

Analysis of electron microscopic images was performed using the software Fiji [49].

### Cell culture and CRISPR/Cas9

266–6 cells were cultivated in Dulbecco’s modified Eagle medium containing 10% fetal calf serum (FCS) and 1% penicillin/streptomycin at 37 °C and 5% CO_2_. Cells were sampled by either using the *QuickExtract™ DNA Extract Solution* (Biozym, Hessisch Oldendorf, Germany) according to the manufacturer’s instruction for DNA extraction or by washing once in ice-cold PBS and resuspension in fresh PBS for protein analysis. 

Cell proliferation was analyzed using the *Cell counting kit—8* (CCK8) from GLPBIO (Montclair, USA) according to the manufacturer’s instructions. ER-stress induction was analyzed using an ER-stress reporter plasmid modified by Iwawaki et al*.* [51]. Therefore, cells were seeded in a 96-well plate and cultivated at 37 °C and 5% CO_2_ until they reached a confluency of 80%. Prior to the transfection with 0.2 µg DNA per well using *Lipofectamine™ 2000* (Thermo Fisher Scientific), the media was changed to DMEM +2% FCS. After 6 h at 37 °C and 5% CO_2_, the media was replaced by DMEM +10% FCS and 1% penicillin/streptomycin. ER-stress was induced 48 h after transfection by adding 6.38 µM CCK or 2 mM DTT to the wells. Analysis of GFP expression was performed before ER-stress induction and after 24 h and 48 h using a FluoStar Optima fluorometer (λ_ex_ = 485 nm, λ_em_ = 520 nm).

For the inactivation of *Cln8* in 266–6 cells, three crRNAs were designed using the Custom ALT-R® CRISPR-Cas9 guide RNA Tool of Integrated DNA Technologies (Coralville, USA) targeting exon 2 of the *Cln8 gene*. All chemicals, RNAs, and transfection reagents were obtained from DNA Integrated Technologies. For transfection, cells were counted using the Casy 1 Model TT, and 150.000 cells were seeded per well in a 24-well plate. The single crRNAs (100 µM) and tracrRNA (100 µM) were incubated in Nuclease-free Duplex Buffer. Then 1 µM ALT-R *S.p.* Cas9 Nuclease V3 was added to the RNA mix and incubated for 5 min at room temperature. Transfection of the cells with the CRISPR/Cas9 mix was performed using *Lipofectamine™ RNAiMax*.

The media was changed after 24 h. After an additional 24 h, cells were resuspended in PBS. Half of the cells were passaged and the other half were used for DNA extraction. The cells were seeded in 96-well-plates by the limiting dilution method. Introduced loss-of-function variants in exon 2 of the *Cln8* gene were confirmed by Sanger sequencing. Deficiency of the CLN8 protein was verified by Western Blot of cell lysates using a CLN8-specific primary antibody. Furthermore, possible off-target effects of the crRNAs were ruled out by Sanger sequencing.

For additional materials and methods see supplementary materials.

## Supplementary Information

Supplementary Material 1.

## Data Availability

The authors declare that all supporting data of this study are provided within the figures and the supplementary information. If any raw data or further information is needed, they are available from the corresponding author upon request.
