# Supplementary material for: Loss of the ER-cargo protein CLN8 increases severity of acute pancreatitis and upregulates ER-stress and ER-phagy
Source: Mol Biomed. 2026 Jun 15;7:91. doi: 10.1186/s43556-026-00479-4 (PMC13269577; doi:10.1186/s43556-026-00479-4)
Supplement: Supplementary file 1 — Supplementary Material 1. [file 43556_2026_479_MOESM1_ESM.docx]

**Loss of the ER-cargo protein CLN8 increases severity of acute pancreatitis and upregulates ER-stress and ER-phagy**

Lukas Zierke^1^, Marcel Gischke^1^, Matthias Sendler^1^, Frank Ulrich Weiss^1^, Silvia Ribback^2^, Dariush Skowronek^3^, Matthias Rath^3,4^, Henry Völzke^5^, Markus M. Lerch^6^, Ali A. Aghdassi^1^

*^1^ Department of Medicine A, University Medicine Greifswald, Greifswald, Germany.*

*^2^ Department of Pathology, University Medicine Greifswald, Greifswald, Germany.*

*^3^ Department of Human Genetics, University Medicine and Interfaculty Institute of genetics and Functional Genomics, University of Greifswald, Greifswald, Germany.*

*^4^ Institute for Molecular Medicine, MSH Medical School Hamburg, Hamburg, Germany.*

*^5^ Institute for Community Medicine, University Medicine Greifswald, Greifswald, Germany.*

*^6^ Ludwig-Maximilians University Munich, Munich, Germany.*

**Corresponding author:**

Ali A. Aghdassi, MD

Department of Medicine A

University Medicine Greifswald

Ferdinand-Sauerbruch Str.

17475 Greifswald

Germany

E-Mail: ali.aghdassi@med.uni-greifswald.de

**Supplementary methods:**

**Materials:**

Collagenase from the bacteria *Clostridium histolyticum* was purchased from Serva (14007). Enterokinase (E0885), Cholecystokinin (CCK) (C-9901), 4′,6-Diamidine-2′-phenylindole dihydrochloride (DAPI), propidium iodide (p4170), morin hydrate (M4008-2G), menadion (M5625) and caerulein (C9026) were purchased from Sigma Aldrich. The substrates for fluorometric activity measurement of cathepsin B (AMC-Arg2, 4004789) and cathepsin L (AMC-Phe-Arg, 4003379) were from Bachem. The trypsin substrate R110-Ile-Pro-Arg (10208) was obtained from BioTrend and Boc-Gln-Ala-Arg-AMC (BML-P237-0005) from Enzo Life Science. Anesthesia of mice was done with ketamine and xylazine, both from Selectavet. Measurement of ATP concentration was performed using the ATP assay kit (ab83355) from Abcam. Tetramethylrhodamine (TMRM) and CellROX Deep Red (C10422) were purchased from Invitrogen, and Carbonyl Cyanide Chlorophenylhydrazone (CCCP) and Sytox Deep Red from Thermo Fisher Scientific. E-64-d (sc-201280) was obtained from Santa Cruz Biotechnology.

**Antibodies:**

The following primary antibodies were used for immunoblot analysis, co-immunoprecipitation, and immunofluorescence labeling: anti-amylase (dilution: 1:100, sc-46657, Santa Cruz, RRID:AB_626668), anti-ATF6 (dilution: 1:1000, ab37149, Abcam, RRID:AB_725571), anti-BIP (dilution: 1:1000 (western blot) and 1:100 (immunofluorescence), 3183, Cell Signaling Technology, RRID:AB_10695864), anti-Calreticulin (dilution: 1:1000, 2891, Cell Signaling Technology, RRID:AB_2275208), anti-CTSB (dilution: 1:1000 (western blot) MAB965, R&D systems, RRID:AB_2086935; dilution: 1:100, AF965, R&D systems), anti-CTSC (dilution: 1:1000, sc-5647, Santa Cruz Biotechnologies, RRID:AB_2086961), anti-CTSD (dilution: 1:1000 (western blot) and 1:100 (immunofluorescence), sc-6486, Santa Cruz Biotechnologies, RRID:AB_637896), anti-CCPG1 (dilution: 1:1000, 13861-1-AP, Proteintech Group Inc., RRID:AB_2074010), anti-CHOP (dilution: 1:1000 (Western Blot) and 1:100 (immunofluorescence), MA1-250, Invitrogen, RRID:AB_2292611), anti-CLN8 (dilution: 1:1000 (western blot) and 1:100 (immunofluorescence), bs-11715R, Bioss Inc.), anti-FAM134B (dilution: 1:1000 (western blot) and 1:100 (immunofluorescence), E8Y9R, Cell Signaling Technology), anti-GAPDH (dilution: 1:1000, H86501M, Meridian Bioscience, RRID:AB_151542), anti-GM130 (dilution: 1:100, 12480S, Cell Signaling Technology), anti-LAMP-1 (dilution: 1:50, sc-20011, Santa Cruz Biotechnologies, RRID:AB_626853), anti-LAMP-2 (dilution: 1:100, PA1-655, Invitrogen), anti-LC3 (for Western Blot: dilution: 1:1000, 2775, Cell Signaling Technology, RRID:AB_915950; for immunofluorescence: dilution: 1:100, 3868, Cell Signaling Technology, RRID:AB_2137707), anti-LIMPII (dilution: 1:1000, NB400-129, Novus Biologicals, RRID:AB_2301298), anti-P62 (dilution: 1:1000, P0067, Sigma Aldrich, RRID:AB_1841064), anti-SEC62 (dilution: 1:1000, A303-981A, Bethyl Laboratories, RRID:AB_2620330), anti-syncollin (dilution: 1:1000 (western blot) and 1:100 (immunofluorescence), ab178415, Abcam), anti-trypsin (dilution: 1:1000, sc-137077, Santa Cruz Biotechnology, RRID:AB_2300318), anti-α/β-tubulin (dilution: 1:1000, 2148S, Cell Signaling Technology, RRID:AB_2288042).

Secondary antibodies were anti-mouse IgG-Alexa 594-conjugated (dilution: 1:200, A-21203, Invitrogen), anti-mouse IgG-Alexa Plus 488-conjugated (dilution: 1:200, A32723, Invitrogen), anti-mouse IgG-Cy3-conjugated (dilution: 1:200, Jackson Immunoresearch), anti-rabbit IgG-Alexa 488-conjugated (dilution: 1:200, A-11008, Invitrogen), anti-rabbit IgG-Cy3-conjugated (dilution: 1:200, Jackson Immunoresearch), anti-rabbit IgG-Cy5-conjugated (dilution: 1:200, Jackson Immunoresearch), anti-goat IgG-Cy3 (dilution: 1:200, Jackson Immunoresearch), anti-goat IgG-Cy5 (dilution: 1:200, A21447, Invitrogen), anti-goat IgG-HRP (dilution: 1:16000, sc-2020, Santa Cruz Biotechnologies), anti-mouse IgG-HRP (dilution: 1:16000, NA931, Amersham plc), anti-rabbit IgG-HRP (dilution: 1:16000 (Western Blot) and 1:200 (Immunofluorescence), NA934V, Amersham plc), anti-rat IgG-HRP (dilution: 1:16000, HAF005, R&D systems).

**Nucleotides:**

For CRISPR/Cas9, the following oligonucleotides were used: 5’-GAAGAUUCGGUCGACUCUAGCGGGUUUUAGAGCUAUGCU-3’, 3’-UCUUCCACGAAGCGUAGUCCAGGGUUUUAGAGCUAUGCU-5’, 5’-CUUUGUCGGCAUAGAGCACGGGGGUUUUAGAGCUAUGCU-3’.

The following primers were used for qPCR analysis (all in 5’-3’ orientation): GCCCGATCTCGTCTGATCTC (5S_fwd), GCCTACAGCACCCGGTATTC (5S_rev), CTCATCAAGATAATCAGACGGCG (Map1lc3b_fwd), TCCCGAATGTCTCCTGCG (Map1cl3b_rev), ACTACGACCTGTGCAGCG (Sqstm1_fwd), AGCTGTAGGGCAAGGGCG (Sqstm1_rev), TGGATAAGAGAGAGGGAGAGAAG (Hspa5_fwd), CACCACTTCAAAGACACCATTG (Hspa5_rev), CCAGGAAACGAAGAGGAAGAA (Ddit3_fwd), TTTGGGATGTGCGTGTGA (Ddit3_rev), GAAAGTTCCAGCGACAGCG (Ccpg1_fwd), ACTGACGATGTGTTGTTTGCG (Ccpg1_rev), GGCGACACAAGAAGCG (Sec62_fwd), ACTCCCTGGTTGTAAATAAAGCG (Sec62_rev), TCTCACTTCCTCTCAAGAGCG (Fam134b_fwd), ATGGCAGCCGTCATGGCG (Fam134b_rev), GAGCAGACTCCCAGTTCTCG (Pink1_fwd), GTCCCACTCCACAAGGATGT (Pink1_rev), ATACCATGAAGGGGATTGCG (Prkn_fwd), AGCGAGCTTGCTCAGCG (Prkn_rev), GGGGAAGAATTGAAAGTGAAGGC (Atg3_fwd), CTCCATCTGTTTACACCGC (Atg3_rev), TATCAGACCACGACGGAGCG (Atg5_fwd), TCTCTCCATCTTCAGGGGCG (Atg5_rev), AAGCTGATGTGCTGGCG (Atg7_fwd), GGAGTAGGAGATCTTGGCG (Atg7_rev), CAGGTCGTTTTTGCCTAGGC (Atg10_fwd), CATTCATCGTTCACTAAAGCG (Atg10_rev), TACTTACCACAGCCCAGGCG (Becn1_fwd), AGAGACACCATCCTGGCG (Becn1_rev), GGGATGCCTTTGTGGAACTA (Becn2_fwd), TGAGCAGGGTCTTCAGAGA (Becn2_rev), CTAGAAACCCCGAAACCAAA (mtDNA_fwd), CCAGCTATCACCAAGCTCGT (mtDNA_rev), ATGGGAAGCCGAACATACTG (totalDNA_fwd), CAGTCTCAGTGGGGGTGAAT (totalDNA_rev).

**mRNA expression analysis**

RNA was extracted from acinar cells by TRIzol (15596026, Invitrogen) according to the manufacturer’s protocol. cDNA was obtained using the High-Capacity cDNA Reverse Transcription Kit (4374966, Applied Biosystems) like suggested by the company. The PowerTrack SYBR Green mastermix (A46111, Applied Biosystems) was used for the qPCR analysis.

**Western Blot and immunoprecipitation:**

Western Blot and SDS-PAGE were performed as described previously (1). Samples were transferred to a nitrocellulose membrane by using a blotting buffer according to Towbin (2). Blocking of the membrane was done using NET buffer (0.15 M sodium chloride, 5 mM EDTA, 50 mM Tris-HCL, 0.05% Triton-X100) that contains 0.2% gelatine. The primary antibodies were diluted 1:1000 and the secondary antibodies 1:16000 in the blocking buffer. The membranes were developed by using the Super Signal West Femto Chemiluminescence kit or the Super Signal Pico Chemiluminescence kit (Thermo Scientific, Waltham, USA). Quantification of bands was performed using Fiji (3).

For immunoprecipitation, rProtein A Sepharose^TM^ Fast Flow and Protein G Sepharose^TM^ Fast Flow (both GE Healthcare, Chicago, USA) were preincubated with the prey antibody for 1 h at room temperature on a rolling incubator. Thereafter, the beads were washed three times with PBS and incubated with the samples and 10 µl 1 M nafamostat. Samples were incubated at the rolling incubator at 4 °C overnight. On the next day, samples were washed 5 times with PBS. After the last washing step, the pellets were resuspended in 2x Laemmli-buffer, heated up to 95 °C for 5 min, and directly used for SDS-PAGE and Western Blot.

**Human sample collection**

EDTA blood samples from chronic pancreatitis patients were collected at the university medicine Greifswald after approval of institutional review board (III UV 05/06). Control blood was drawn from healthy volunteers of the Study of Health in Pomerania (SHIP), a population-based study in northeastern Germany (4). The presented SHIP groups were sub-cohorts of the SHIP-TREND cohort, in which 4420 participants were examined between 2008 and 2012 (BB 39/08). All blood donors gave written and informed consent. Human chronic pancreatitis tissues were collected in the context of the ChroPac trial (ISRCTN38973832). The *Cln8* gene was analyzed by whole genome sequencing of the human blood samples (Centogene, Rostock, Germany). The prediction of the functional effect of the SNPs was performed using PolyPhen-2 (5).

**Statistics:**

All statistical analyses were performed using GraphPad Prism (GraphPad Software, Boston, USA, RRID: SCR_002798). The data were presented as mean +/- standard error. Normal distribution of results was tested with the Shapiro-Wilk test. For statistical analysis between two groups of normally distributed data, the unpaired Student’s *t-test*, and of non-normally distributed data or low sample numbers, the Mann-Whitney test was performed. For testing of statistical difference between more than two groups, the ordinary one-way ANOVA with Tukey’s multiple comparisons test was used for normally distributed data and the Kruskal-Wallis test with Dunn’s multiple comparisons test for non-normally distributed data. Differences were considered statistically different at a level of p < 0.05.

**References:**

1. Aghdassi AA, John DS, Sendler M, Weiss FU, Reinheckel T, Mayerle J, et al. Cathepsin D regulates cathepsin B activation and disease severity predominantly in inflammatory cells during experimental pancreatitis. J Biol Chem. 2018 Jan 1;293(3):1018–29. doi:10.1074/jbc.M117.814772

2. Towbin H, Staehelin T, Gordon J. Electrophoretic transfer of proteins from polyacrylamide gels to nitrocellulose sheets: procedure and some applications. Proc Natl Acad Sci U S A. 1979 Jan 1;76(9):4350–4. doi:10.1073/pnas.76.9.4350

3. Schindelin J, Arganda-Carreras I, Frise E, Kaynig V, Longair M, Pietzsch T, et al. Fiji: an open-source platform for biological-image analysis. Nat Methods. 2012 Jan 1;9(7):676–82. doi:10.1038/nmeth.2019

4. Volzke H, Alte D, Schmidt CO, Radke D, Lorbeer R, Friedrich N, et al. Cohort Profile: The Study of Health in Pomerania. International Journal of Epidemiology. 2011 Apr 1;40(2):294–307. doi:10.1093/ije/dyp394

5. Adzhubei IA, Schmidt S, Peshkin L, Ramensky VE, Gerasimova A, Bork P, et al. A method and server for predicting damaging missense mutations. Nat Methods. 2010 Apr;7(4):248–9. doi:10.1038/nmeth0410-248


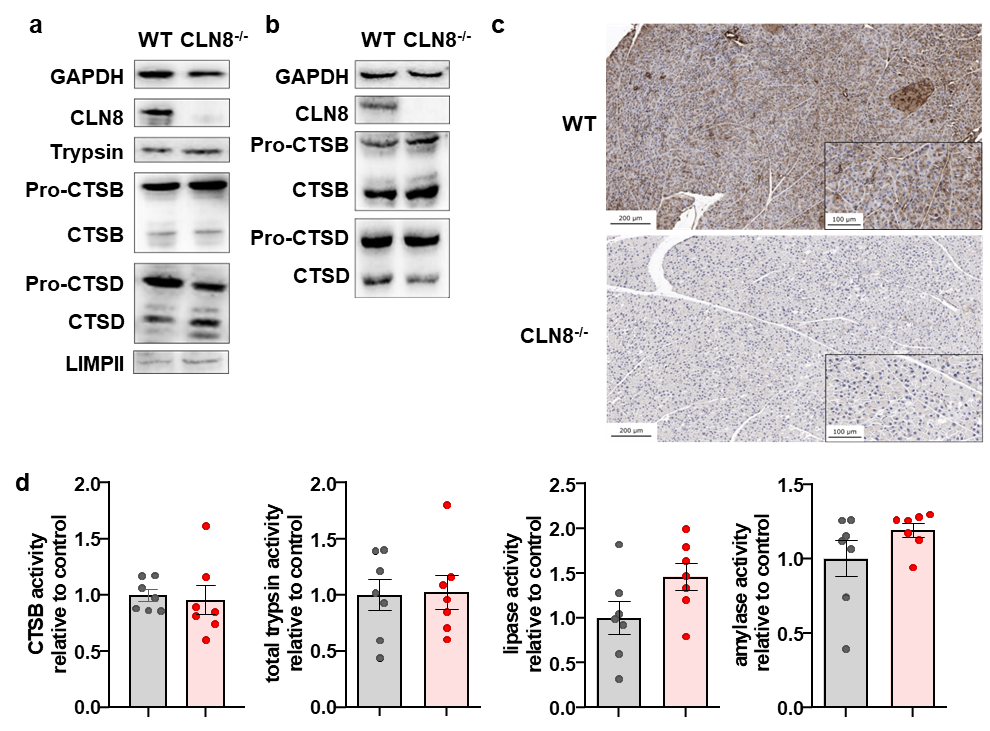


**Fig. S1:** Characterization of the pancreas and liver of CLN8-deficient mice. **a:** Immunoblot analysis of pancreas homogenates of unstimulated CLN8-competent wild type mice (WT) and CLN8-deficient mice (CLN8^-/-^) showed no differences in trypsin, cathepsin B (CTSB), cathepsin D (CTSD), and LIMP-II expression despite absence of CLN8. **b.** Immunoblot analysis of liver homogenates of WT and CLN8-deficient mice showed no differences in CTSB and CTSD expression. **c:** Immunohistochemical staining in paraffin-embedded slides of pancreatic tissue confirmed CLN8 knockout in CLN8-deficient mice. Gross histology was normal. **d:** CTSB, lipase and amylase activity as well as total trypsin activity after enterokinase incubation in pancreas homogenates displayed no differences despite CLN8 knockout. At least six animals were used for these experiments and the measurements were performed in triplicates. Values are mean +/- SEM. * denotes p < 0.05

**
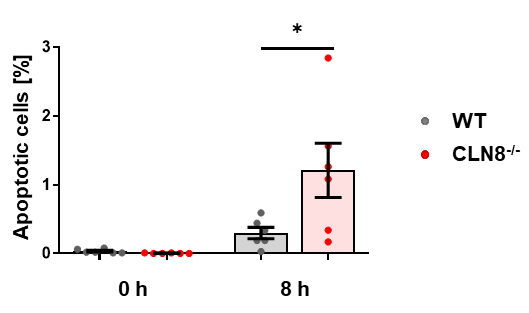
**

**Fig. S2:** Tunel staining of paraffin-embedded pancreatic tissues of WT and CLN8-deficient mice at 0 h and 8 h acute pancreatitis followed by quantification showed an increased number of apoptotic cells at 8 h acute pancreatitis in the CLN8^-/-^ mice. Six animals were used for this experiment. Values are mean +/- SEM. * denotes p < 0.05.

**
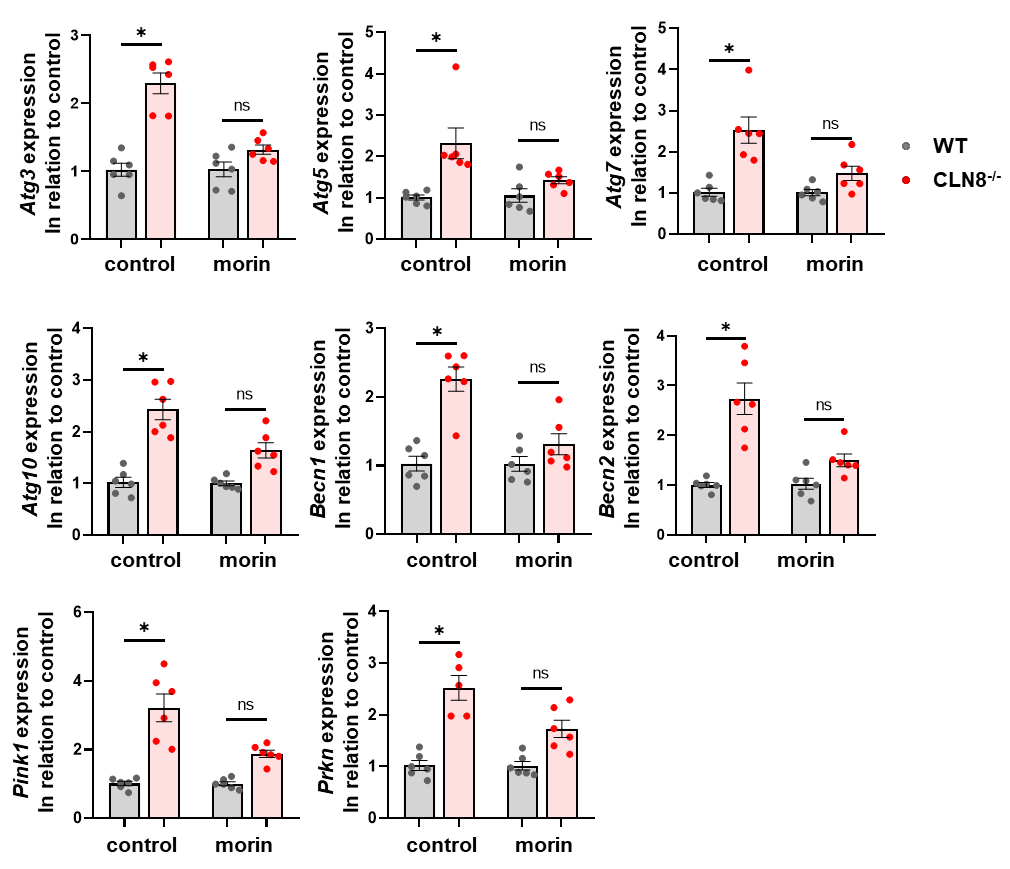
**

**Fig. S3:** Analysis of mRNA expression by qPCR of acinar cells, illustrated a significantly upregulated expression of the autophagy-related genes *Atg3, Atg5, Atg7, Atg10, Becn1,* and *Becn2* in CLN8-deficient acinar cells (CLN8^-/-^) compared to WT. Similar results were obtained for the mitophagy-related genes *Pink1* and *Prkn*. Addition of morin markedly reduced the mRNA expression in CLN8-deficient cells. This reduction was much stronger than in wild types. Six animals were used for each experiment and measurements were performed in triplicates. Values are mean +/- SEM. * denotes p < 0.05.


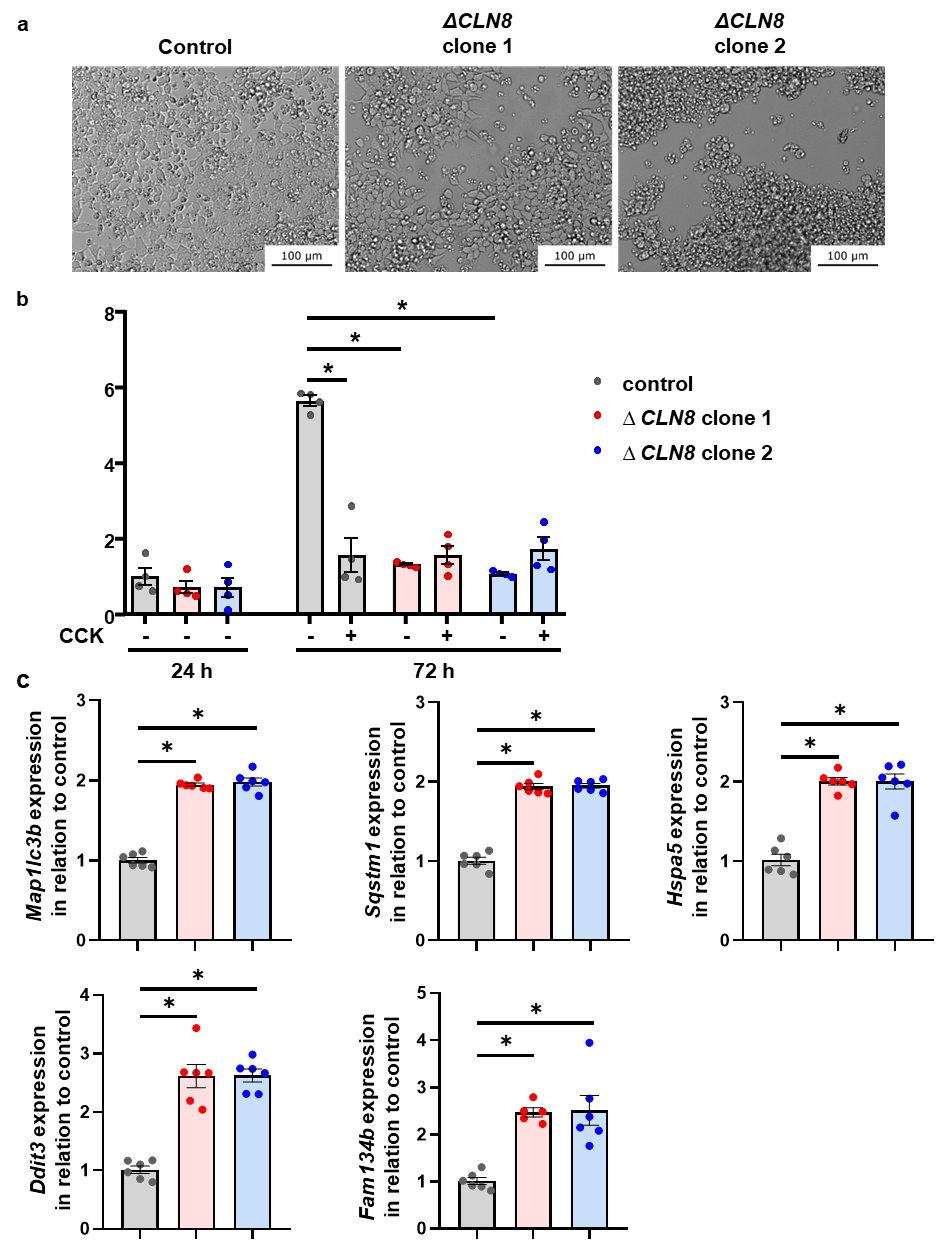


**Fig. S4:** Morphological and mRNA expression analysis of CLN8^-/-^ 266-6 cells. **a:** CLN8-deficient cell clones 266-6 ΔCLN8 1 and ΔCLN8 2 generated by CRISPR/Cas9 were characterized by an abnormal cellular morphology and a more clustered proliferation. **b:** Quantification of CCK-8 intensities as a marker of cell proliferation showed a clearly lower proliferation in CLN8-deficient cell clones at 72 h after dispersion of cells. While control cells responded with a significant decrease in cell proliferation upon CCK-stimulation, no significant differences were noted for the CLN8-deleted clones. Equal numbers of cells were seeded. **c.** The mRNA expression of the *Map1lc3b, Sqstm1, Hspa5, Ddit3,* and *Fam134b* genes were significantly upregulated in CLN8-deficient cells compared to controls. The values represent the results of at least four independent experiments and the measurements were performed in duplicates. Values are mean +/- SEM. * denotes p < 0.05.


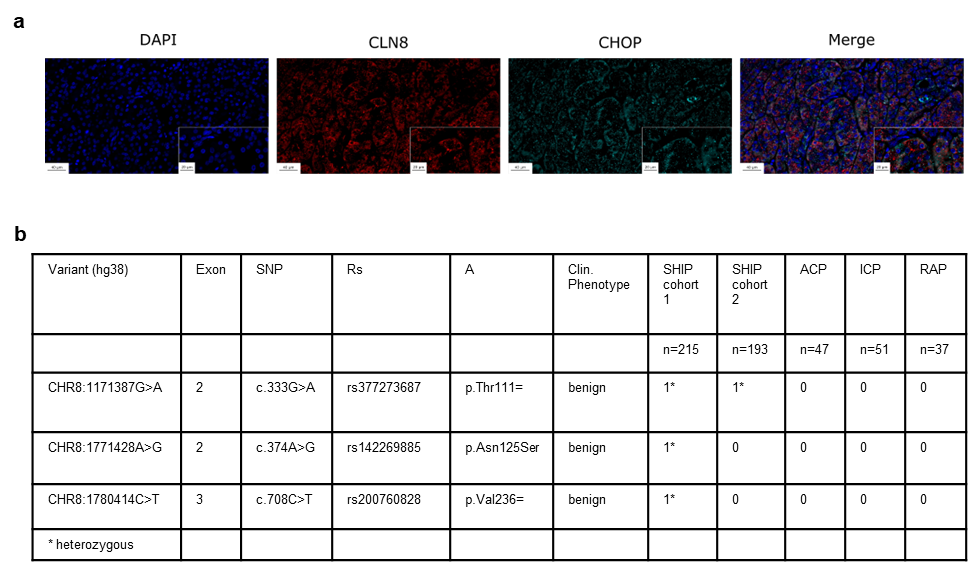


**Fig. S5:** Analysis of CLN8 expression and mutations in humans. **a.** CLN8 is expressed in the human pancreas. Chronic pancreatitis tissues were labelled for CLN8 (red). Co-labeling for CHOP (cyan) indicated presence of ER-stress in human chronic pancreatitis. Representative images from immunofluorescence staining of six patients with chronic pancreatitis. **b.** Whole genome sequencing of the *Cln8* gene in two cohorts of a population-based study (Study of Health in Pomerania, SHIP cohort 1 and 2) and in patients with alcoholic chronic pancreatitis (ACP), idiopathic chronic pancreatitis (ICP), and recurrent acute pancreatitis (RAP) identified three single nucleotide polymorphisms (SNPs) in the *Cln8* gene. All variants were found in the SHIP cohorts. No variants were found in patients with ACP, ICP, and RAP. Two SNPs (p.Thr111=; p.Val236=) were coding for the same amino acid and were therefore silent. The SNP p.Asn125Ser was a missense mutation and led to an amino acid exchange. The functional effects of each variant were assessed by PolyPhen-2 software and were classified as benign.
